# Comparative study of the rotating bending fatigue performance of wire arc additively manufactured hollow shafts with and without TIG pre-joining

**DOI:** 10.1371/journal.pone.0357916

**Published:** 2026-09-18

**Authors:** Van-Minh Nguyen, Pham Son Minh, Tran Minh The Uyen, Hai Hung Pham

**Affiliations:** 1 Ho Chi Minh City University of Technology and Engineering, Ho Chi Minh City, Vietnam; 2 University of Labour and Social Affairs, Ha Noi, Vietnam; King Mongkut’s University of Technology North Bangkok, THAILAND

## Abstract

This study investigates the effect of Tungsten Inert Gas (TIG) pre-joining on the rotating bending fatigue performance of hollow shafts fabricated by Wire Arc Additive Manufacturing (WAAM) from ER70S-6 wire deposited onto stacked CCT34 low-carbon steel substrate rings. A four-factor, five-level Response Surface Methodology with Central Composite Design was employed across 30 runs, with welding current, bead overlap, deposition speed, and ring thickness as independent variables and fatigue life as the response. Analysis of variance confirmed that all four parameters and their quadratic terms significantly influenced fatigue life (*p* < 0.001), with deposition speed and welding current dominant. The optimal parameter set predicted a fatigue life of 505,427 cycles. Validation specimens (*n* = 5) achieved a mean fatigue life of 490,973 cycles under a bending stress amplitude of 208.5 MPa (*R* = −1), approximately 50% higher than WAAM-only specimens (*n* = 5, mean = 327,344 cycles). Weibull analysis indicated lower scatter for the TIG pre-joined configuration and a 62.9% higher B10 characteristic life, although the limited sample size means these estimates are preliminary. Metallographic sectioning revealed that WAAM deposition alone left an unfused region extending 0.175 mm from the bore at each ring-to-ring interface, whereas TIG pre-joining achieved complete penetration through the full ring wall. Microhardness profiling showed a hardened zone confined to the interface in the TIG pre-joined condition, 10.6 HV0.3 above the corresponding location in WAAM-only specimens (*p* = 0.004), while the two configurations were indistinguishable away from the interface (*p* = 0.85). Microscopy further showed a finer, more uniform polygonal ferrite structure with single-site crack initiation in the TIG pre-joined condition, against coarser and more widely distributed grain sizes with multi-site initiation in WAAM-only specimens. TIG pre-joining is therefore an effective strategy for enhancing both the fatigue life and the reproducibility of WAAM hollow shafts.

## 1. Introduction

Wire Arc Additive Manufacturing (WAAM) is a directed energy deposition (DED) process that uses an electric arc as the heat source and metallic wire as feedstock to build near-net-shape components layer by layer [1,2]. Compared to powder-bed fusion techniques, WAAM offers significantly higher deposition rates, lower material waste, and lower equipment cost, making it particularly attractive for medium-to-large structural components [3]. In recent years, WAAM has been applied to a variety of engineering applications including aerospace frames, ship propellers, and automotive components [4,5]. Hollow shafts with integrated internal cooling channels represent a critical component class in high-performance rotating machinery, where weight reduction and thermal management are simultaneously required [6]. Conventional manufacturing of such geometries, involving deep drilling, gun drilling, or welded assemblies, is time-consuming and cost-prohibitive, particularly for small-batch production [7]. WAAM on stacked substrate ring preforms offers an alternative route, in which the internal geometry is predefined by the arrangement of the substrate rings before deposition [8].

A fundamental challenge in this approach is achieving adequate metallurgical bonding between adjacent substrate rings and the deposited WAAM bead. Without a pre-joining step, the interface between stacked rings may contain gaps, oxide layers, and inadequate fusion, which can act as fatigue crack initiation sites [8,9]. Tungsten Inert Gas (TIG) welding is recognised as a low heat input process that offers superior arc stability and precise control over welding parameters compared with other arc welding techniques [10]. These characteristics allow accurate regulation of heat input, making TIG particularly suitable as a pre-joining method to consolidate the stacked substrate ring assembly prior to WAAM deposition. Circumferential TIG passes can eliminate inter-ring gaps and oxide layers, thereby providing a continuous and metallurgically sound substrate for subsequent WAAM layers. Specimens fabricated using this approach are subjected to two successive thermal cycles. The first arises from the initial TIG pre-joining pass, which establishes a heat-affected zone at the inter-ring interfaces [11]. The second is imposed by the multi-layer WAAM deposition, during which each subsequent layer reheats the material beneath it [12]. Depending on the peak temperature and cooling rate, the secondary thermal cycle may promote grain refinement through intercritical reheating (Ac1–Ac3 range) and partial recrystallisation rather than excessive coarsening, resulting in a more homogeneous microstructure that is beneficial for fatigue performance [13,14]. The sensitivity of weld microstructure and properties to the imposed thermal cycle is well documented across steel systems. In austenitic stainless steel weldments, post-weld thermal exposure governs carbide precipitation and the resulting degree of sensitisation, with measurable consequences for microhardness and corrosion resistance [49] and for the metallurgical condition of both weld metal and heat-affected zone [50]. Of more direct relevance to the present work, Kumar et al. [51] showed that applying a secondary GTA remelting pass to an arc-deposited layer modified the microstructure of the deposit and raised its microhardness — a thermal sequence analogous in principle to the TIG-plus-WAAM route examined here. Although the controlling metallurgical mechanism differs in low-carbon steels, where the relevant transformation is ferrite–pearlite rather than carbide precipitation, these studies establish the general principle that a secondary thermal cycle applied to arc-deposited material can substantially alter its microstructure and mechanical performance.

Fatigue failure under rotating bending is one of the most common failure modes for rotating shafts in service [15,16]. The influence of welding process parameters on fatigue performance has been extensively studied for conventionally welded joints [17,18], but systematic investigation of the combined effects of WAAM process parameters on rotating bending fatigue life, particularly in combination with a pre-joining step, remains limited. Previous studies have focused predominantly on static mechanical properties such as tensile strength and hardness [19,20], leaving the fatigue behaviour of WAAM components on structured substrates undercharacterised. Response Surface Methodology (RSM) with Central Composite Design (CCD) is a well-established statistical framework for process optimisation involving multiple interacting variables [21]. It enables quantitative modelling of complex non-linear relationships between process parameters and performance metrics. Its application to arc-based processes is well established, spanning conventional weld bead geometry optimisation and prediction of mechanical properties in MIG/MAG joints [22], and more recently the optimisation of deposition parameters in WAAM [23,24], where the number of interacting variables makes one-factor-at-a-time experimentation impractical [25].

Efforts to improve the fatigue performance of WAAM components have to date followed three broad routes: post-deposition thermal treatment to homogenise the as-deposited microstructure; mechanical surface treatments such as shot peening, laser peening, or machining, which introduce compressive residual stress and remove as-built waviness; and in-process interventions such as interpass rolling or ultrasonic peening, which refine the solidification structure layer by layer. What these approaches share is that they operate on a monolithic deposit built on a simple flat or bar-shaped substrate. The fatigue-critical feature they address is therefore internal to the deposit itself, whether porosity, columnar grain morphology, or surface waviness. The configuration examined here differs in kind rather than in degree. Building a hollow shaft on a stack of discrete substrate rings introduces a fatigue-critical feature that does not exist in monolithic WAAM at all, namely a series of transverse ring-to-ring interfaces distributed along the axis of the component. Because deposition proceeds from the outer surface inward, the root of each interface lies at the bore and is the last region to be reached by the molten pool, so that any incompleteness in fusion is concentrated precisely where the process has least control. Neither post-deposition heat treatment nor surface treatment can address a discontinuity of this type, because it lies beneath the deposit and is created before deposition begins. It must instead be eliminated at the assembly stage, which is the specific function of the TIG pre-joining step investigated here.

Existing fatigue studies of WAAM low-carbon steel do not cover this configuration. Ermakova et al. [28] characterised both uniaxial and multiaxial fatigue of WAAM ER70S-6 but tested solid bar specimens extracted from a monolithic wall, and Fang et al. [26] examined high-cycle fatigue of WAAM carbon steel plates; in both cases the specimen contained no substrate interface [27]. Studies of hollow or tubular WAAM geometries have concentrated on deposition strategy and dimensional accuracy rather than on cyclic performance, and investigations of WAAM on structured substrates have reported static properties such as tensile strength and hardness [29,30] without extending to fatigue. Consequently, no quantitative data exist on how WAAM process parameters govern the rotating bending fatigue life of a hollow shaft built on a segmented substrate, nor on the magnitude of the improvement attainable by consolidating that substrate before deposition. This study addresses that gap. Its specific objectives are to (1) establish a quantitative RSM-CCD model relating four WAAM process parameters to the rotating bending fatigue life of hollow shafts built on TIG pre-joined substrate ring assemblies; (2) quantify the fatigue life and life-scatter penalty incurred when the pre-joining step is omitted, at otherwise identical optimal parameters; and (3) identify the mechanisms responsible for the difference through metallographic sectioning, optical microscopy, microhardness profiling, and SEM fractography.

## 2. Materials and methods

### 2.1 Materials

Substrate rings were fabricated from low-carbon steel CCT34 with an inner bore diameter of 10 mm and outer diameter of 14.5 mm at the gauge section, with ring thickness varying according to the experimental design (1 mm to 5 mm as specified in Table 2). Dimensional tolerances were maintained below 0.1 mm. Multiple rings were coaxially stacked and clamped using an M10 threaded rod passed axially through the assembly to form the deposition preform. The WAAM filler material was ER70S-6 (EN ISO 14341-A G 42 3M21 3Si1) wire with diameter 1.2 mm, a copper-coated low-alloy steel wire conforming to AWS A5.18 with excellent deposition characteristics and good wetting behaviour [31]. Argon shielding gas (purity ≥ 99.99%) was supplied at a flow rate of 12 L/min for both TIG and WAAM processes [32,33]. Nominal chemical compositions of the two materials are given in Table 1.

**Table 1:** Nominal chemical composition (wt %) of the ER70S-6 filler wire and the CCT34 substrate ring material.

Material	C	Si	Mn	P	S	Fe
ER70S-6	0.06–0.15	0.80–1.15	1.40–1.85	≤ 0.025	≤ 0.035	Bal.
CCT34	0.09–0.15	0.12–0.30	0.25–0.50	≤ 0.040	≤ 0.050	Bal.

*Compositions are nominal values specified by the respective standards (AWS A5.18 / EN ISO 14341-A for ER70S-6; TCVN 1765−75 for CCT34) and were not measured on the actual material heats used.*

The material pairing was selected on metallurgical, functional, and practical grounds. Because the bond between the deposited bead and the substrate ring is formed by arc melting and solidification, the interface is metallurgically a fusion weld rather than a coating or a mechanical joint, and its integrity is governed by the same factors that govern weldability in conventional arc welding of low-carbon steels: compatibility of chemical composition, carbon equivalent, and solidification range between the two materials. ER70S-6 and CCT34 are both low-carbon, low-alloy steels with closely comparable carbon and manganese contents, so that dilution at the interface produces a fusion zone whose composition lies between two similar parent compositions rather than spanning a dissimilar-metal gap. This avoids the hard martensitic transition layers, sharp hardness discontinuities, and differential thermal contraction that arise when dissimilar alloy systems are combined, all of which are known to promote interfacial crack initiation under cyclic loading.

ER70S-6 was further selected because its elevated silicon and manganese content provides strong deoxidation, which is important when depositing onto a ground ring surface that may retain a thin oxide film, and because it exhibits good wetting and bead-forming characteristics across a wide current range — a prerequisite for the broad parameter window explored in this study [31]. From a practical standpoint, ER70S-6 in 1.2 mm diameter is the most widely available and lowest-cost solid welding wire in the Vietnamese market and is compatible with standard MIG power sources, while CCT34 seamless tube is a commodity product available in the ring dimensions required. Since the objective of this work is to establish a manufacturing route for hollow shafts with internal cooling channels that can be adopted using existing workshop equipment, the use of commodity consumables rather than specialised alloys is a deliberate design choice rather than a limitation.

The mating faces of the substrate rings were finish-turned to a controlled surface texture of *R*_a_ 1.6 µm rather than being polished or left as-cut. This value was selected deliberately to ensure that the interfacial contact condition, and hence the residual micro-gap between adjacent rings under clamping, was reproducible across all ring pairs and all experimental runs. Because the ring-to-ring interface is the feature under investigation, holding its initial condition constant was a prerequisite for attributing differences in fatigue life to the deposition parameters and to the presence or absence of TIG pre-joining, rather than to variability in the as-machined interface.

### 2.2 Equipment

**Fig 1:**
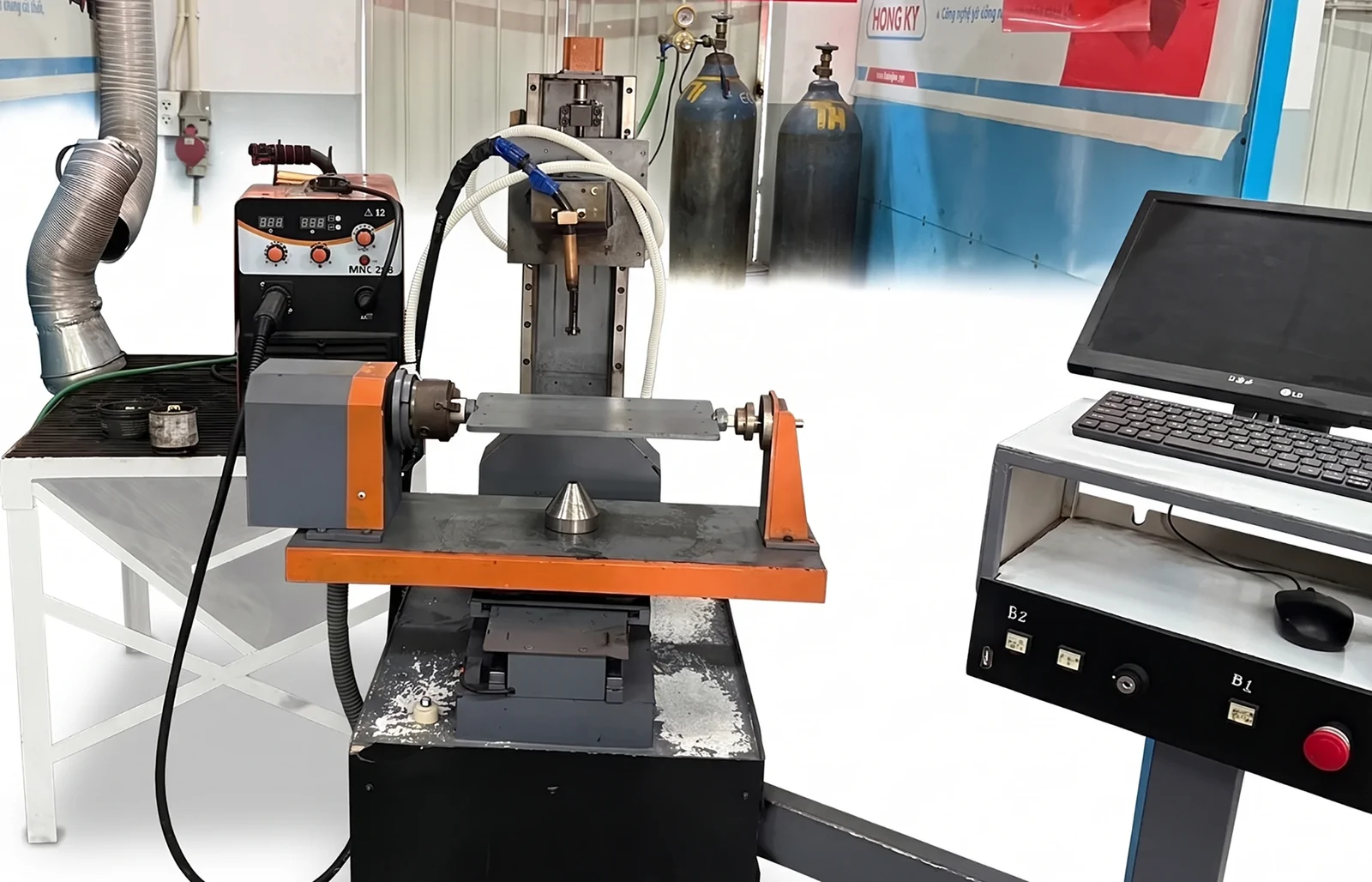
WAAM 4-axis TEA CNC system setup.

TIG pre-joining was performed using a Jasic TIG200 power source. WAAM deposition was carried out using a Jasic MIG250 power source mounted on a 4-axis TEA CNC system as illustrated in Fig 1, enabling fully automated circumferential deposition. Fatigue testing was conducted on a TESCA rotating bending fatigue machine. All specimens were machined to comply with ISO 1143 [34] standard geometry, with an outer diameter of 18 mm and inner bore of 10 mm at the test section.

Surface roughness of the machined fatigue specimens was measured using a Mitutoyo SJ-210 portable surface roughness tester to ensure consistent surface quality prior to fatigue testing. Microstructural examination was performed using an Olympus BX51 upright reflected-light materials microscope for optical microscopy (OM) observations. Vickers microhardness measurements were performed using a Mitutoyo HM-101 microhardness tester (Mitutoyo, Tokyo, Japan). Fractographic analysis of the fatigue fracture surfaces was conducted with a HITACHI TM4000 tabletop scanning electron microscope (SEM) operating at an accelerating voltage of 15–20 kV.

**Fig 2:**
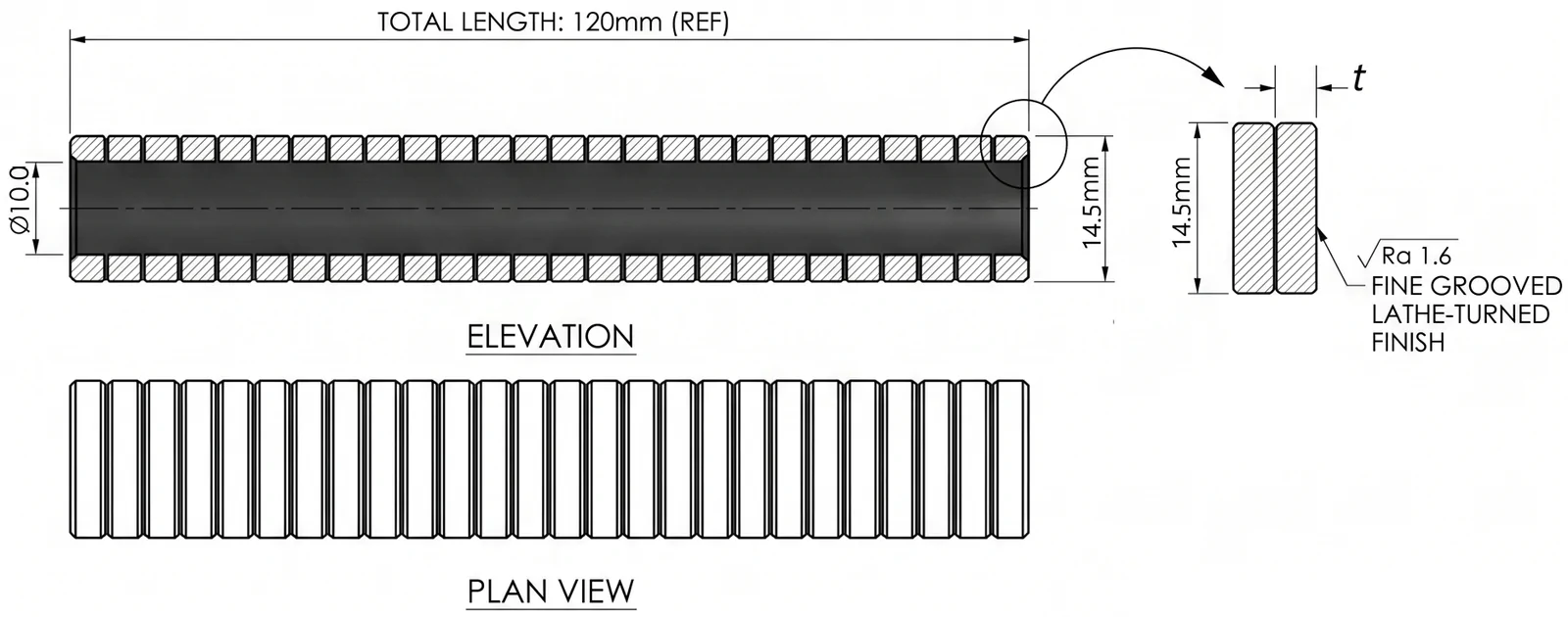
Schematic of the CCT34 low-carbon steel substrate ring stack assembly used as the deposition substrate. Dimensions in mm. The 120 mm stack length is nominal; where the required length was not an integer multiple of the ring thickness, an additional ring was included and the assembly machined back symmetrically after deposition.

**Fig 3:**
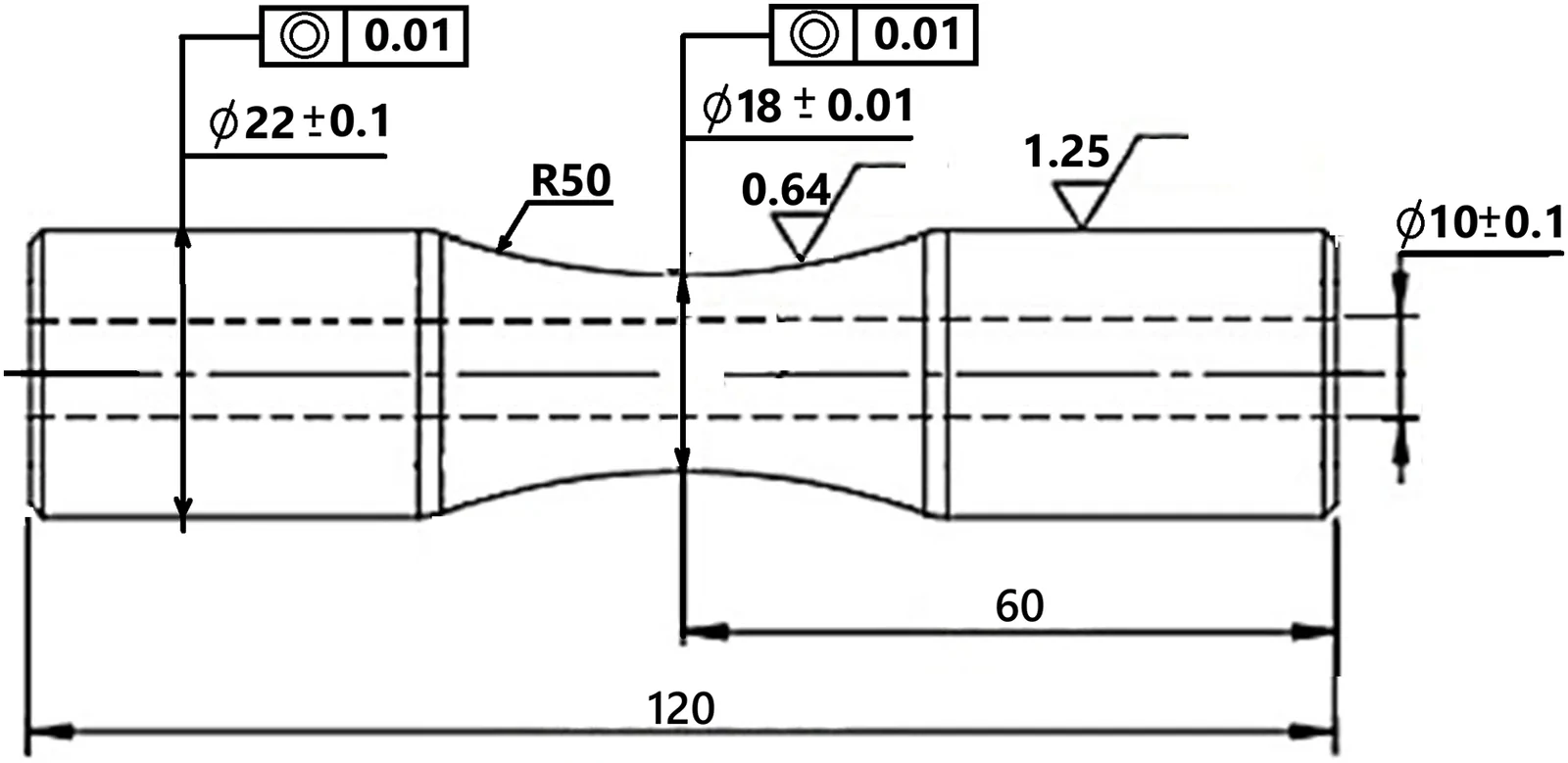
Rotating bending fatigue specimen geometry conforming to ISO 1143 [34]. All dimensions in mm.

The detailed geometries of the substrate ring and the standard fatigue specimen are presented in Fig 2 and Fig 3, respectively.

### 2.3 TIG pre-joining parameters

Based on literature values for low heat input arc welding of low-carbon steels and preliminary trials, TIG pre-joining parameters were fixed as follows: welding current *I*_tig_ = 70 A, arc length *L* = 2 mm, welding speed *v*_tig_ = 80 mm/min, and bead overlap *O*_tig_ = 2 mm. These parameters were deliberately chosen to maintain a low heat input regime (estimated at approximately 0.25–0.35 kJ/mm, assuming typical TIG arc voltage of 10–12 V and thermal efficiency *η* ≈ 0.6–0.7) [35,36]. Low heat input is critical because it promotes rapid cooling rates, resulting in a finer grain structure in the heat-affected zone (HAZ) while still providing sufficient fusion to eliminate gaps and oxide layers between the stacked substrate rings [1,2]. Previous studies have consistently shown that increasing heat input leads to significant grain coarsening in the HAZ of low-carbon steels, with average grain size rising markedly, which negatively affects mechanical properties and fatigue resistance [11,14,30]. Conversely, low heat input regimes help minimise grain growth, reduce the width of the HAZ, and improve microstructural homogeneity [37,38].

In this work, the selected combination of moderate current (70 A) and relatively high travel speed (80 mm/min) ensures adequate penetration at the inter-ring interfaces without excessive heat accumulation or distortion. Circumferential TIG passes were applied around the full periphery of the stacked ring assembly using the automated 4-axis CNC system.

### 2.4 WAAM process and experimental design

Four WAAM process parameters were identified as independent variables based on their known influence on deposition quality, interlayer bonding, and mechanical properties [25,39]: welding current (*I*), bead overlap (*O*), deposition speed (*v*), and substrate ring thickness (*t*).

The selected ranges for these parameters (Table 2) were determined from preliminary experiments, manufacturer recommendations for the ER70S-6 wire with 1.2 mm diameter, and the fundamental heat input relationship in MIG welding. Heat input was estimated using Eq (1):

**Table 2:** WAAM process parameter levels for the RSM-CCD experimental design.

Coded Level	Current *I* (A)	Overlap *O* (mm)	Speed *v* (mm/min)	Ring Thickness *t* (mm)
−α	110	2.0	400	1
−1	115	2.25	450	2
0 (Center)	120	2.5	500	3
+1	125	2.75	550	4
+α	130	3.0	600	5


$$\begin{array}{c} HI=\frac{\eta \times U\times I}{v\times \text{1}\text{0}\text{0}\text{0}/\text{6}\text{0}}\; \end{array}$$ (1)

where *HI* is the heat input (kJ/mm), *η* ≈ 0.75 is the typical arc efficiency for MIG welding on steel, *U* is the arc voltage (V), *I* is the welding current (A), and *v* is the travel speed (mm/min). In the present study, the arc voltage was held constant at 20 V for all experimental runs. This value was determined through a series of preliminary exploratory trials conducted prior to the main RSM-CCD campaign, in which arc voltage was systematically varied between 18 V and 24 V while all other parameters were held at their centre-point values. Arc stability was assessed by visual inspection of the arc behaviour and spatter pattern, together with examination of the resulting bead morphology. A voltage of 20 V was found to consistently yield a stable, low-spatter arc and a continuous, well-formed bead across the full range of welding current (110–130 A) and travel speed (400–600 mm/min) combinations investigated. Voltages below this threshold produced an irregular, stubbing arc, while higher voltages resulted in increased spatter and irregular bead profiles. Accordingly, 20 V was adopted as a fixed parameter to isolate the effects of the four primary variables under investigation. The chosen parameter ranges correspond to an estimated heat input from approximately 0.18 kJ/mm (high speed, low current) to 0.45 kJ/mm (low speed, high current). This range was intentionally designed to cover both insufficient fusion conditions and excessive heat accumulation leading to coarse columnar grains, which are commonly reported in WAAM of low-carbon steels. Such a broad parameter window allows the Response Surface Methodology to capture the main effects, quadratic effects, and interactions among the variables.

A four-factor, five-level RSM-CCD was designed, yielding 30 experimental runs comprising 16 factorial points, 8 axial (star) points, and 6 centre-point replicates. The factor levels and coded values are summarised in Table 2.

Substrate ring assemblies were built to a nominal stack length of *L*_s_ = 120 mm, which is independent of the loading arm *L* used in the fatigue test (Section 2.5). The number of rings in each assembly was therefore determined by the ring thickness under investigation, ranging from 120 rings at *t* = 1 mm to 24 rings at *t* = 5 mm. Where the required stack length was not an integer multiple of the ring thickness, an additional ring was included and the assembly was machined back symmetrically from both ends after deposition to the nominal length. For the validation configuration (*t* = 3.6 mm), 34 rings were stacked to give 122.4 mm and 1.2 mm was removed from each end. Because the ring count remained even in every configuration and any excess material was removed symmetrically, a ring-to-ring interface coincided with the mid-span of the assembly, and hence with the minimum cross-section of the finished fatigue specimen, in all 30 RSM runs and in all validation specimens. This represents the most severe possible placement of the interface, positioning it where the bending stress is greatest, and the measured fatigue lives therefore characterise the worst-case geometry rather than a favourable one.

After TIG pre-joining, the stacked substrate ring assemblies were subjected to stress-relief annealing in an electric resistance furnace according to ISO 17663 [40]. The thermal cycle consisted of heating at a controlled rate not exceeding 100 °C/h to 600 ± 10 °C, soaking for 2 hours, followed by furnace cooling to below 200 °C. This treatment was performed to relieve residual stresses induced by the TIG pre-joining process without significantly altering the microstructure.

Subsequently, the annealed substrate assemblies underwent concentric grinding to achieve a precise outer diameter of 14 mm. The ground surfaces were then carefully inspected to ensure the absence of macroscopic defects such as protrusions or depressions. Surface roughness was measured using a Mitutoyo SJ-210 tester and maintained at *R*_a_ ≈ 0.25 µm to provide a consistent surface for WAAM deposition.

Substrate ring assemblies for the WAAM-only configuration were prepared identically to the TIG pre-joined assemblies with respect to concentric grinding to Ø14 mm and surface finish (*R*_a_ ≈ 0.25 µm), so that both groups presented an equivalent surface condition to the deposition process. The intermediate stress-relief anneal was applied only to the TIG pre-joined assemblies, since it serves to relieve residual stresses introduced by the TIG pass and has no counterpart in the WAAM-only route, where the rings are held solely by mechanical clamping prior to deposition.

Following WAAM deposition, all specimens underwent a post-deposition stress-relief annealing using the same thermal cycle. Both groups therefore received an identical final thermal treatment, so that the residual stress state at the time of fatigue testing was governed by the same thermal cycle in both cases.

**Fig 4:**
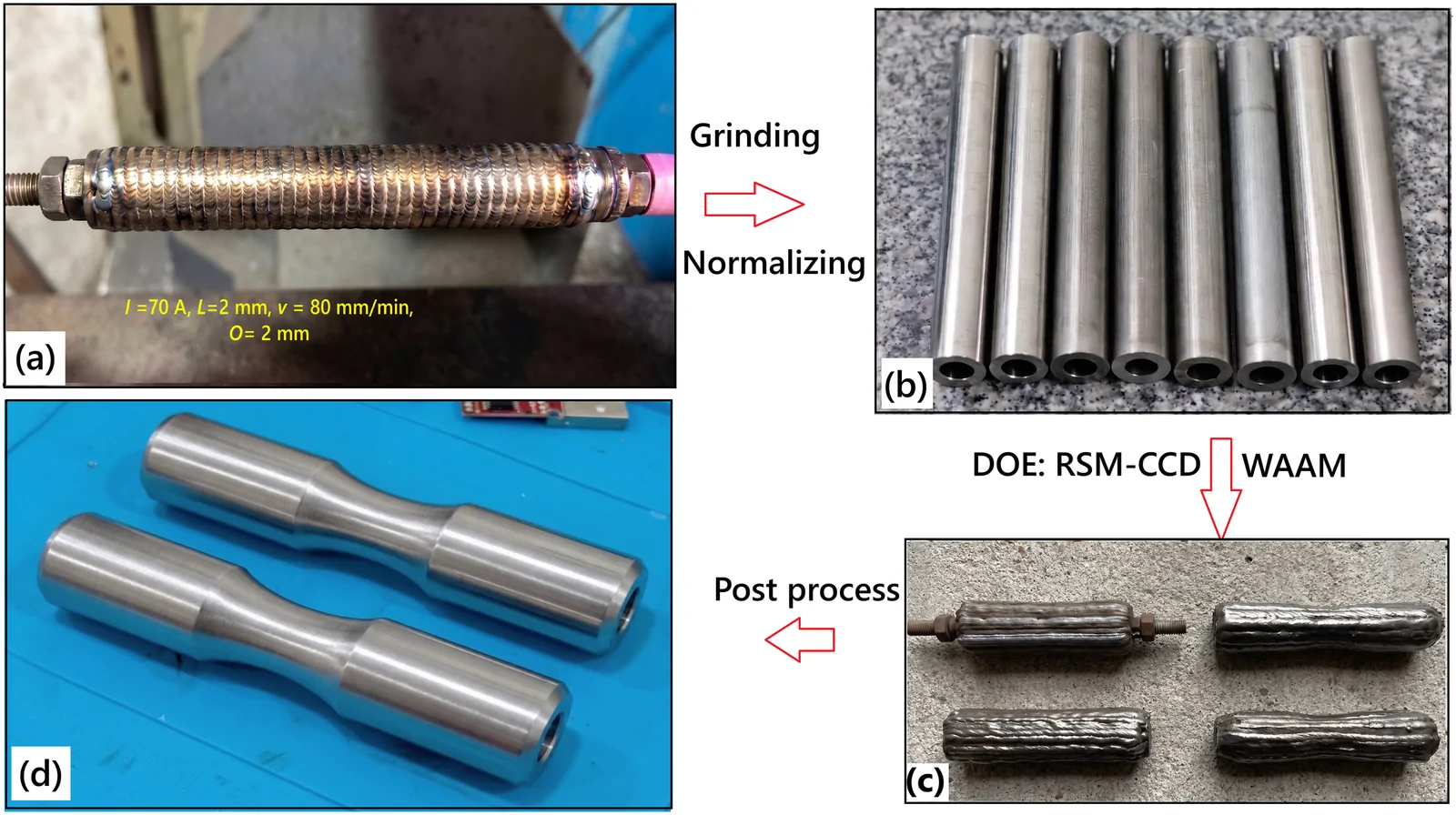
Experimental process sequence: (a) automated CNC TIG pre-joining, (b) after surface preparation, (c) after WAAM deposition, (d) final machined fatigue specimen.

The complete experimental workflow from TIG pre-joining to the final machined fatigue specimen is illustrated in Fig 4.

### 2.5 Fatigue testing

**Fig 5:**
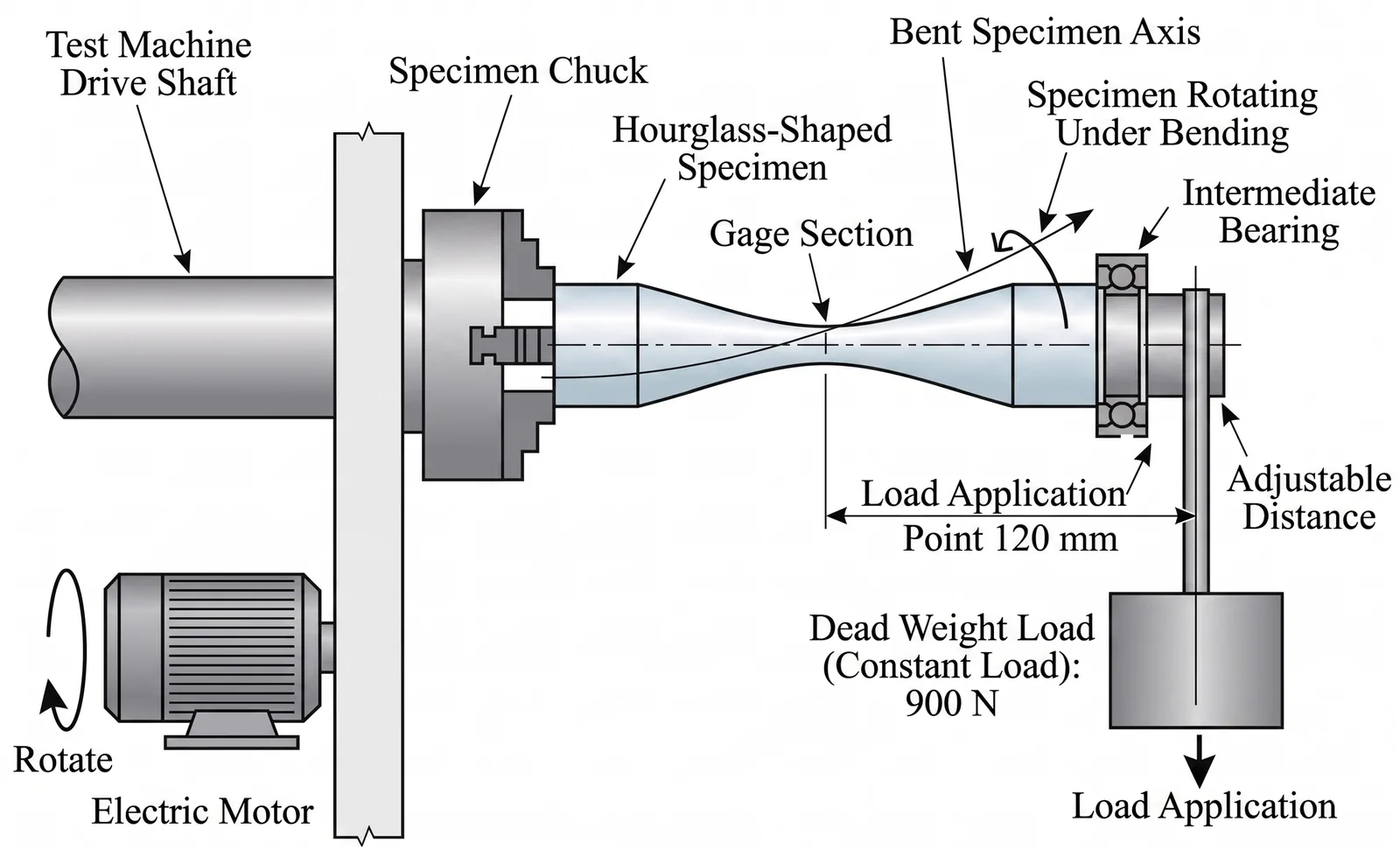
Schematic illustration of the rotating bending fatigue test principle and the applied cyclic stress waveform (*R* = −1).

The principle of the rotating bending fatigue test setup, including the loading configuration and the resulting cyclic stress waveform (*R* = −1), is schematically illustrated in Fig 5. Rotating bending fatigue tests were performed on a TESCA rotating bending fatigue machine at a rotational speed of 2900 rpm. One end of the specimen was firmly clamped to the rotating spindle, while a constant load was applied to the free end through an intermediate bearing. The loading point was positioned at a fixed distance of 120 mm from the centre of the gauge section. During rotation, the gauge section experienced a fully reversed bending stress cycle with a stress ratio of *R* = −1. All tests were run continuously to failure without interruption.

A constant point load of *F* = 900 N was applied at a distance of *L* = 120 mm from the minimum cross-section of the gauge area. The specimen geometry follows a hollow hourglass profile with an outer diameter (*D*) of 18 mm and an inner diameter (*d*) of 10 mm. The section modulus (*W*_x_) for the hollow gauge section is calculated using Eq (2):


$$\begin{array}{c} W_{x}=\frac{\pi \left(D^{\text{4}}-d^{\text{4}}\right)}{\text{3}\text{2}D}=\frac{\pi \left({\text{1}\text{8}}^{\text{4}}-{\text{1}\text{0}}^{\text{4}}\right)}{\text{3}\text{2}\times \text{1}\text{8}}\approx \text{5}\text{1}\text{8}\;{\text{mm}}^{\text{3}}\;\; \end{array}$$ (2)

The nominal maximum bending stress amplitude (*σ*_a_) induced at the gauge section is determined by Eq (3):


$$\begin{array}{c} \sigma_{a}=\frac{F\cdot L}{W_{x}}=\frac{\text{9}\text{0}\text{0}\times \text{1}\text{2}\text{0}}{\text{5}\text{1}\text{8}}\approx \text{2}\text{0}\text{8}.\text{5}\;\text{MPa}\;\; \end{array}$$ (3)

This stress level corresponds to approximately 46%–60% of the yield strength of the deposited ER70S-6 material (*σ*_y_ ≈ 350–450 MPa [28,41]), which forms the outer 2 mm of the specimen wall and therefore governs behaviour at the outer fibre where the bending stress is greatest and where crack initiation was observed in all specimens. By maintaining the stress amplitude below the yield limit, the specimen operates within the elastic deformation regime, and the recorded lives of 2.9–5.2 × 10^5^ cycles place all tests within the high-cycle fatigue (HCF) regime, well above the conventional 10⁴-cycle boundary for low-cycle fatigue. This setup ensures that the fatigue life is predominantly governed by the crack initiation phase, particularly influenced by the microstructural features and potential defects inherent to the wire arc additive manufacturing process, in accordance with ISO 1143.

Wire arc additive manufacturing produces an as-deposited surface with pronounced inter-bead waviness and a stepped profile, and for components used in the as-built condition this surface morphology is recognised as a dominant factor controlling fatigue life, frequently overshadowing the influence of internal defects or microstructure. In the present study this variable was deliberately eliminated by design rather than left uncontrolled. All specimens, both TIG-WAAM and WAAM-only, were CNC turned and subsequently polished at the gauge section after deposition, removing the entire as-deposited surface layer together with its waviness, and were verified by stylus profilometry (Mitutoyo SJ-210) to satisfy the surface finish requirement of ISO 1143, with measured *R*_a_ values below 0.64 µm. Surface roughness was thus held constant across both configurations at a level where it is not the life-limiting feature, which is a necessary condition for attributing the observed differences in fatigue life to the interfacial and microstructural effects of TIG pre-joining rather than to surface topography. The consequence is that the fatigue lives reported here characterise the machined condition. For as-built WAAM shafts the absolute lives would be considerably lower, and the relative benefit of TIG pre-joining could differ, since surface-initiated failure would compete with the interfacial mechanism identified in this work. Quantifying fatigue performance in the as-deposited surface condition, and determining the minimum machining allowance required to recover the machined-condition life, are identified as directions for further work in Section 4.5.

**Fig 6:**
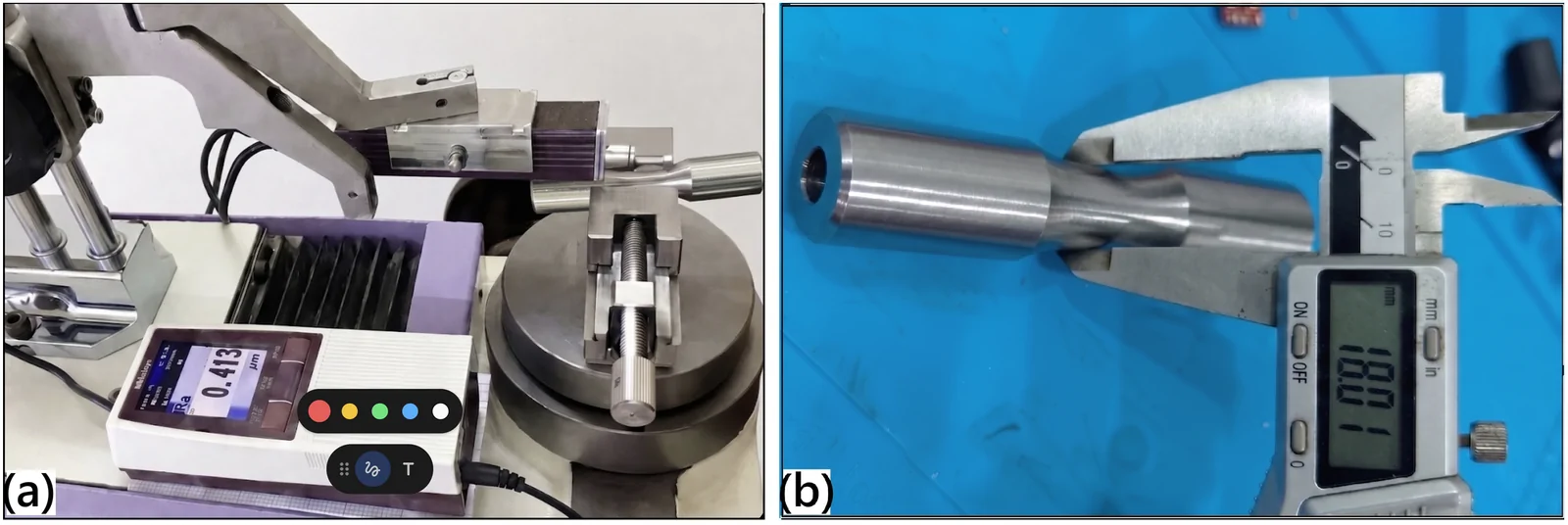
Surface quality verification: (a) roughness measurement setup, (b) completed specimen gauge checked prior to fatigue testing.

**Fig 7:**
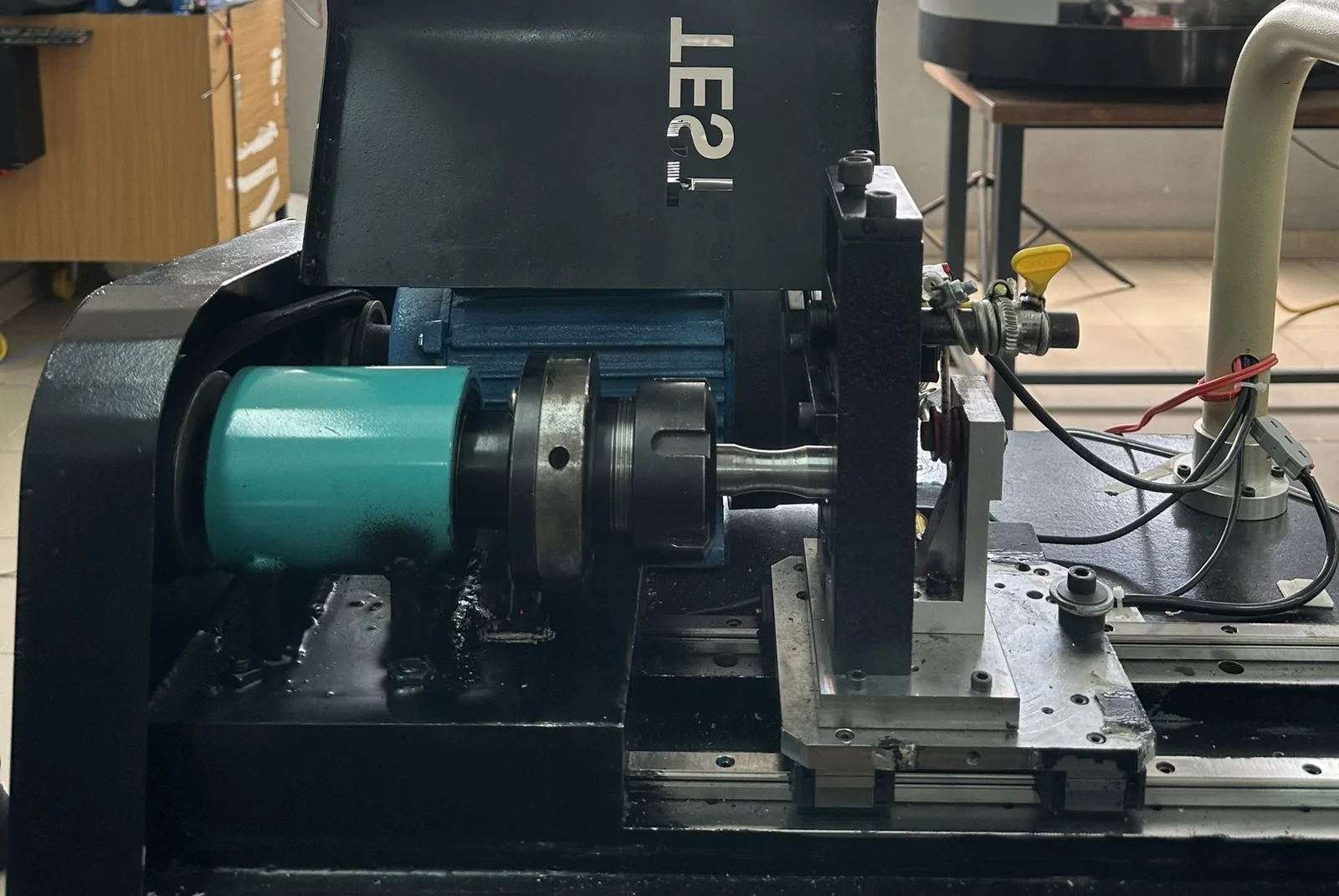
Rotating bending specimen installed on the TESCA fatigue machine.

All tests were conducted in a controlled laboratory environment at 28 ± 2 °C and relative humidity below 60% to minimise the influence of temperature variation and corrosion on fatigue performance. The primary measured response was the number of cycles to failure (*N*_f_). Surface roughness of all machined specimens was measured prior to testing to ensure consistency, as shown in Fig 6. The fatigue testing configuration on the TESCA machine is shown in Fig 7.

### 2.6 Statistical analysis

All experimental data obtained from the 30 runs of the RSM-CCD design were analysed using Minitab 21 statistical software. A second-order polynomial response surface model was fitted to the fatigue life data. Model adequacy was evaluated through several statistical criteria, including the coefficient of determination (*R*^2^), adjusted *R*^2^, predicted *R*^2^, and analysis of variance (ANOVA) at a significance level of α = 0.05.

The optimisation of WAAM parameters to maximise fatigue life within the experimental domain was performed using the desirability function approach. This statistical tool allows simultaneous consideration of multiple responses and provides a composite desirability value that indicates how well the solution satisfies the optimisation goal.

In addition to the descriptive statistical comparison and Welch’s *t*-test, a two-parameter Weibull analysis was performed on the fatigue life data from both validation groups (*n* = 5 per group) to characterise the reliability and scatter behaviour of each configuration. Weibull shape (*β*) and scale (*η*) parameters were estimated by least-squares regression on linearised Weibull probability paper using Benard’s median rank approximation for cumulative failure probability, Eq (4):


$$\begin{array}{c} F(t)=\frac{i-\text{0}.\text{3}}{n+\text{0}.\text{4}}\;\; \end{array}$$ (4)

B10 and B50 characteristic lives, corresponding to 10% and 50% cumulative failure probability respectively, were calculated from the fitted parameters.

### 2.7 Microstructural characterisation

Specimens for microstructural examination were sectioned at the gauge region, mounted in epoxy resin, ground, and polished to a mirror finish of 0.05 µm using standard metallographic procedures. Etching was performed with 2% Nital solution to reveal the microstructure. Optical microscopy was carried out using an Olympus BX51 microscope at magnifications ranging from 50× to 500 × . Longitudinal sections through the gauge region were additionally prepared by wire electrical discharge machining along the axial direction, followed by identical polishing and etching, to assess interfacial fusion quality at the macroscopic scale. Scanning electron microscopy fractography of the fatigue fracture surfaces was performed using a HITACHI TM4000 SEM operating at an accelerating voltage of 15–20 kV.

Grain size was determined by direct measurement of individual grain diameters on calibrated optical micrographs using the image analysis function of the microscope software. For non-equiaxed grains the mean of the major and minor axes was taken as the representative diameter. Measurements were made within the fusion zone of each configuration and are reported as the observed range.

### 2.8 Microhardness measurement

Vickers microhardness was measured on polished longitudinal cross-sections using a Mitutoyo HM-101 microhardness tester at a test load of HV0.3 with a 10 s dwell, in accordance with ISO 6507−1 [52]. Measurements were made on two specimens: the WAAM-only specimen with the shortest fatigue life and the TIG-WAAM specimen with the longest. These are the same specimens used for all other metallographic and fractographic characterisation, so that the evidence presented in Sections 4.1 and 4.2 derives from a consistent pair. They represent the extremes of their respective validation groups rather than the group means, and the profiles are therefore presented as evidence for the operative mechanism rather than as a statistical comparison of the two populations.

Three traverses were made on each specimen at an indentation pitch of 0.190 mm. Two radial traverses ran from near the bore towards the outer surface, spanning *r* = 5.575 mm to *r* = 8.615 mm in 17 indentations and crossing the substrate–deposit boundary at *r* = 7.0 mm. Traverse A1 was positioned on a ring-to-ring interface and traverse A2 at the mid-ring position 1.8 mm away, so that the two differ only in axial position and provide an internal control for the effect of the interface. The axial positions of the interfaces were established from the fracture plane, which by design coincides with an interface, together with the known ring thickness of 3.6 mm. A third traverse, C, ran axially at *r* = 5.345 mm over a span of 5.89 mm in 32 indentations, crossing two consecutive interfaces located at 1.735 mm and 5.335 mm along the traverse. The traverse layout is shown in Fig 18a.

Each traverse was measured once. Because the sectioned specimens were of limited axial extent, replicate traverses at multiple interfaces were not feasible, and the dispersion reported for each region is the standard deviation of the individual indentations within that region. This reflects local microstructural variation in addition to instrument repeatability, the latter being within 2–3% for Vickers testing and small by comparison. It is further noted that at HV0.3 the indentation diagonal for this material is approximately 55–60 µm, comparable to the measured grain size in both conditions, so that individual readings sample only a small number of grains.

## 3. Results

### 3.1 RSM-CCD Results and ANOVA

The fatigue life results from the 30 experimental runs of the RSM-CCD design are summarised in Table 3. The measured fatigue life ranged from 319,406 cycles (Run 22, high deposition speed) to 498,885 cycles (Run 10, elevated current and thicker substrate ring).

**Table 3:** RSM-CCD experimental design matrix and fatigue life results for TIG-WAAM specimens.

Run	*I* (A)	*O* (mm)	*v* (mm/min)	*t* (mm)	Fatigue *N* (cycles)
1	115	2.25	450	2	447,841
2	115	2.25	450	4	465,579
3	115	2.25	550	2	353,973
4	115	2.25	550	4	385,498
5	115	2.75	450	2	424,800
6	115	2.75	450	4	448,677
7	115	2.75	550	2	344,569
8	115	2.75	550	4	363,027
9	125	2.25	450	2	464,156
10	125	2.25	450	4	498,885
11	125	2.25	550	2	430,565
12	125	2.25	550	4	454,297
13	125	2.75	450	2	455,411
14	125	2.75	450	4	456,922
15	125	2.75	550	2	403,100
16	125	2.75	550	4	437,520
17	110	2.5	500	3	349,045
18	130	2.5	500	3	472,242
19	120	2	500	3	471,391
20	120	3	500	3	435,049
21	120	2.5	400	3	463,478
22	120	2.5	600	3	319,406
23	120	2.5	500	1	425,631
24	120	2.5	500	5	458,174
25	120	2.5	500	3	479,191
26	120	2.5	500	3	486,184
27	120	2.5	500	3	472,719
28	120	2.5	500	3	489,009
29	120	2.5	500	3	478,591
30	120	2.5	500	3	481,888

Non-significant interaction terms (*I × O*, *I × t*, *O × v*, *O × t*, *v × t*; *p* > 0.05) were excluded from the final predictive equation to improve parsimony, while retaining all terms in the ANOVA table for completeness. The fitted second-order polynomial model for fatigue life *N* (in cycles) as a function of coded WAAM parameters is expressed by Eq (5):


$$\begin{array}{c} N=\text{4}\text{8}\text{1},\text{2}\text{6}\text{4}+\text{5}\text{0},\text{2}\text{7}\text{4}I-\text{2}\text{0},\text{7}\text{8}\text{8}O-\text{6}\text{3},\text{9}\text{8}\text{9}v+\text{2}\text{0},\text{0}\text{9}\text{0}t-\text{6}\text{8},\text{2}\text{8}\text{7}I^{\text{2}}-\text{2}\text{5},\text{7}\text{1}\text{1}O^{\text{2}}-\text{8}\text{7},\text{4}\text{8}\text{9}v^{\text{2}}-\text{3}\text{7},\text{0}\text{2}\text{8}t^{\text{2}}+\text{4}\text{4},\text{9}\text{8}\text{4}Iv\;\; \end{array}$$ (5)

ANOVA results (Table 4) indicate that the overall model is highly significant (*F* = 58.47, *p* < 0.001) with *R*^2^ = 98.20%, adjusted *R*^2^ = 96.52%, and predicted *R*^2^ = 90.72%, confirming excellent model fit and predictive capability. The lack-of-fit test was non-significant (*p* = 0.113), indicating no systematic departure from the model form.

**Table 4:** Analysis of variance (ANOVA) for the fatigue life response surface model.

Source	DF	Adj SS	Adj MS	F-Value	P-Value
Model	14	65,991,182,587	4,713,655,899	58.47	0.000
Linear	4	44,746,505,410	11,186,626,352	138.77	0.000
*I* (A)	1	15,164,749,908	15,164,749,908	188.11	0.000
*O* (mm)	1	2,592,762,513	2,592,762,513	32.16	0.000
*v* (mm/min)	1	24,567,424,748	24,567,424,748	304.75	0.000
*t* (mm)	1	2,421,568,241	2,421,568,241	30.04	0.000
Square	4	19,149,410,364	4,787,352,591	59.39	0.000
*I*²	1	7,993,988,389	7,993,988,389	99.16	0.000
*O*²	1	1,133,223,344	1,133,223,344	14.06	0.002
*v*²	1	13,121,650,214	13,121,650,214	162.77	0.000
*t*²	1	2,350,452,805	2,350,452,805	29.16	0.000
2-Way Interaction	6	2,095,266,814	349,211,136	4.33	0.010
*I* × *O*	1	10,778,089	10,778,089	0.13	0.720
*I* × *v*	1	2,023,605,240	2,023,605,240	25.10	0.000
*I* × *t*	1	10,230,402	10,230,402	0.13	0.727
*O* × *v*	1	1,284,822	1,284,822	0.02	0.901
*O* × *t*	1	23,663,360	23,663,360	0.29	0.596
*v* × *t*	1	25,704,900	25,704,900	0.32	0.581
Error	15	1,209,216,548	80,614,437	—	—
Lack-of-Fit	10	1,040,176,465	104,017,646	3.08	0.113
Pure Error	5	169,040,083	33,808,017	—	—
Total	29	67,200,399,135	—	—	—

Among the linear terms, deposition speed (*v*) exerted the largest influence on fatigue life (*F* = 304.75, *p* < 0.001), followed by welding current (*I*). All quadratic terms were statistically significant. Among the two-way interactions, only the interaction between current and deposition speed (*I* × *v*) was significant (*F* = 25.10, *p* < 0.001).

**Fig 8:**
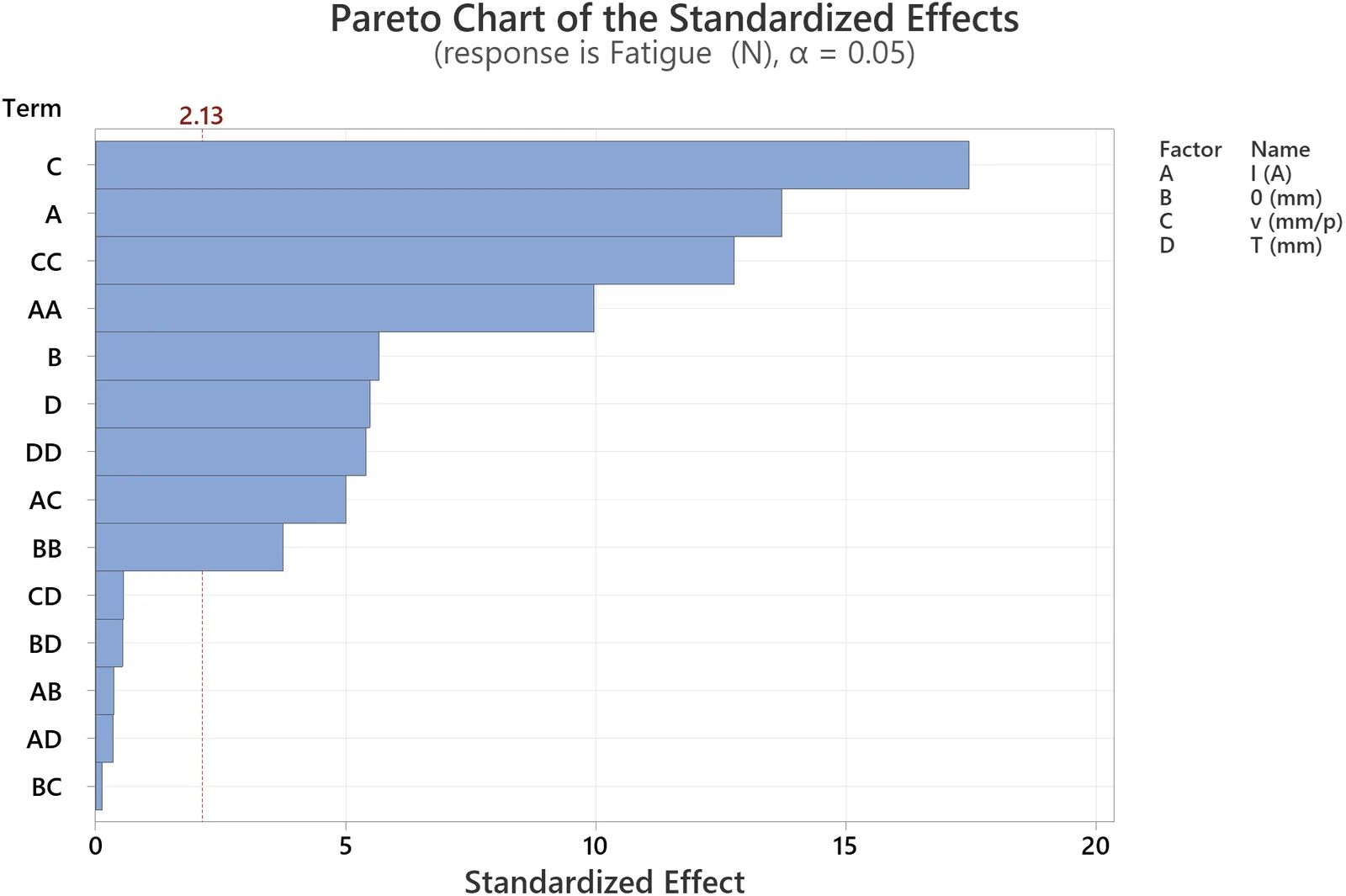
Pareto chart of standardised effects on fatigue life. The reference line at α = 0.05 indicates the significance threshold.

**Fig 9:**
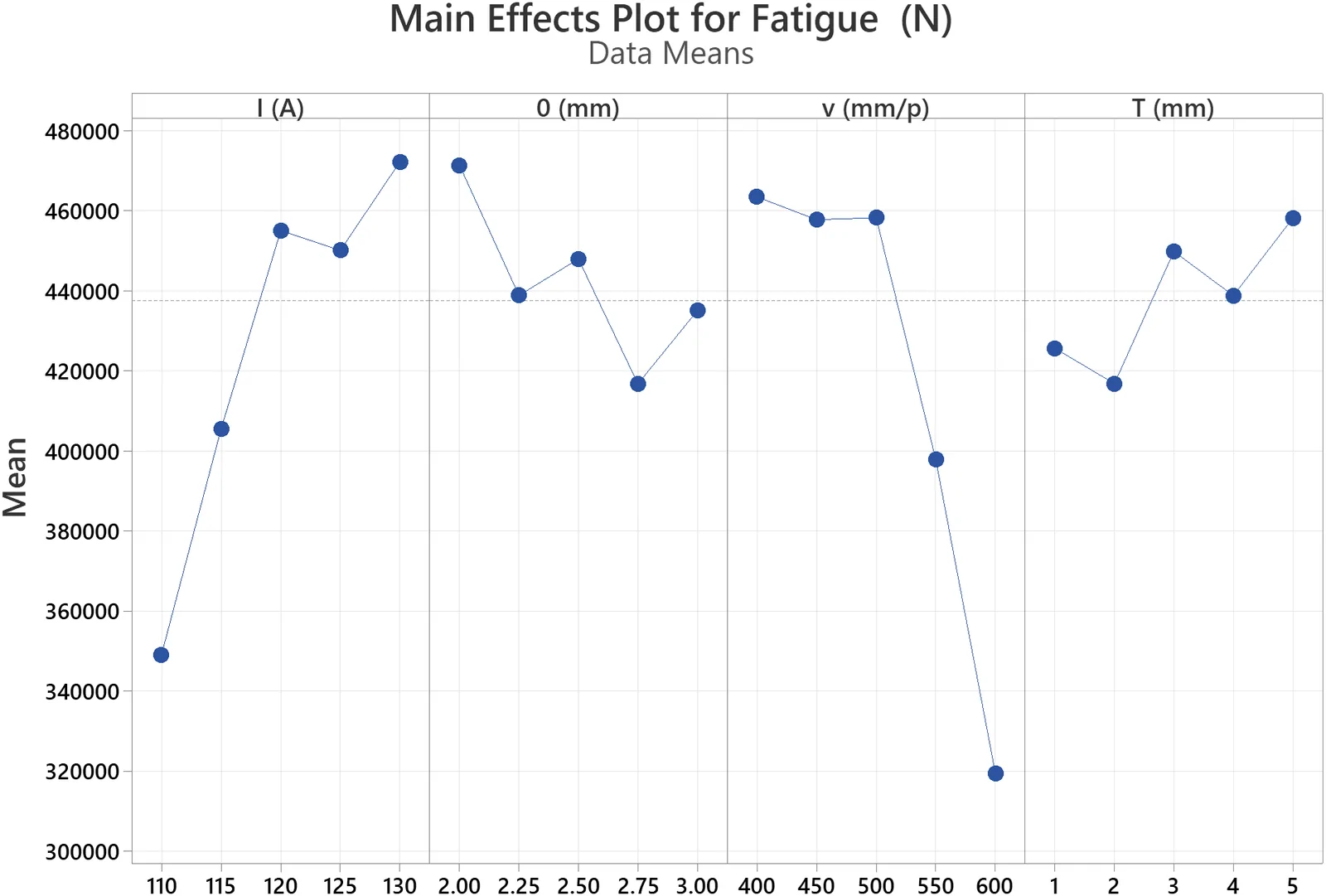
Main effects plot showing the individual influence of each WAAM parameter on mean fatigue life.

**Fig 10:**
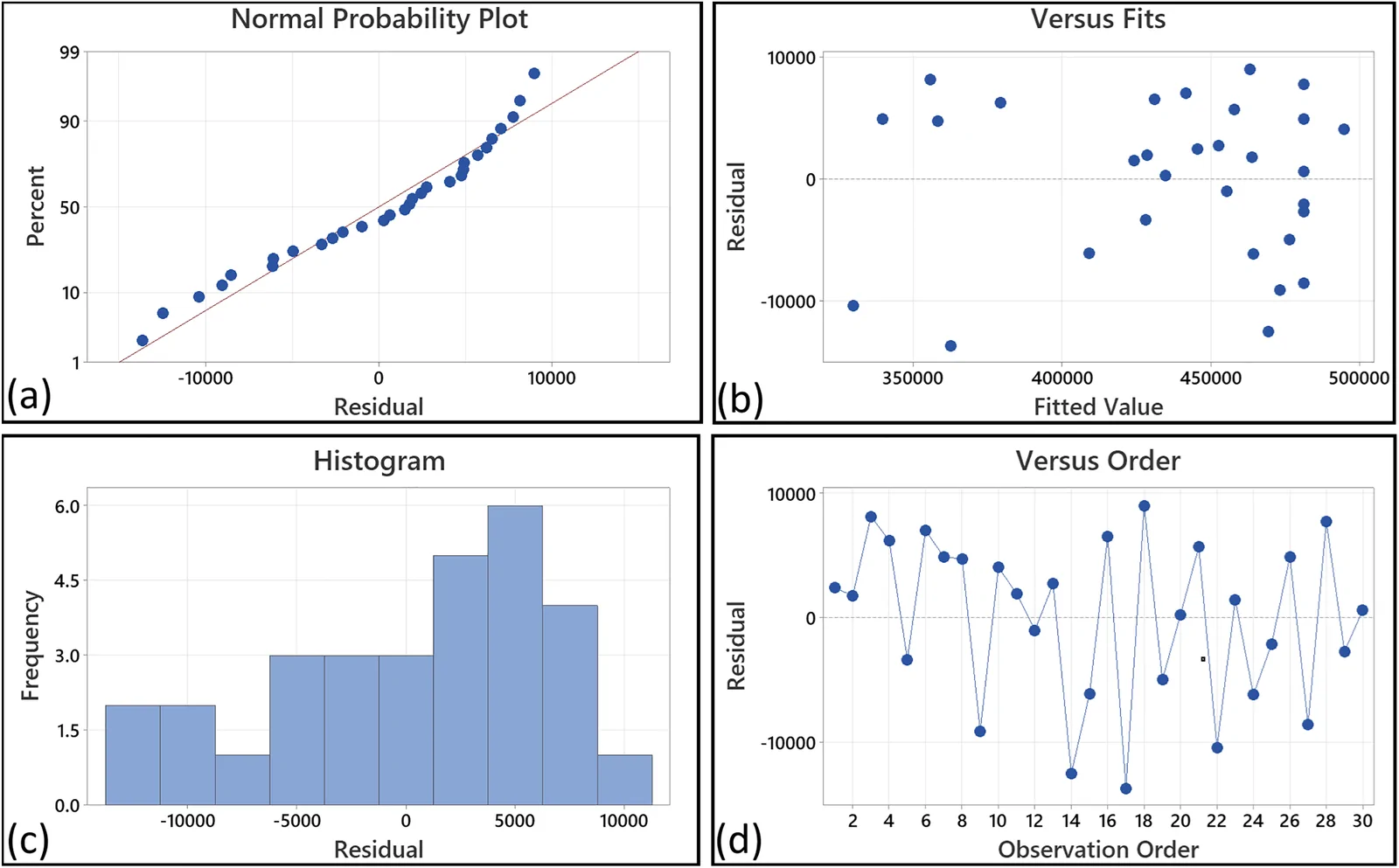
Residual diagnostic plots confirming model adequacy: (a) normal probability plot, (b) residuals versus fitted values, (c) histogram of residuals, (d) residuals versus observation order.

The standardised effects of the parameters are shown in the Pareto chart (Fig 8), while the individual influence of each WAAM parameter on mean fatigue life is illustrated in the main effects plot (Fig 9). Model adequacy was further confirmed by the residual diagnostic plots presented in Fig 10. Coded regression coefficients are given in Table 5.

**Table 5:** Coded regression coefficients for the fatigue life model.

Term	Coef	SE Coef	T-Value	P-Value	VIF
Constant	481,264	3,665	131.30	0.000	—
*I* (A)	50,274	3,665	13.72	0.000	1.00
*O* (mm)	−20,788	3,665	−5.67	0.000	1.00
*v* (mm/min)	−63,989	3,665	−17.46	0.000	1.00
*t* (mm)	20,090	3,665	5.48	0.000	1.00
*I* × *O*	−3,283	8,979	−0.37	0.720	1.00
*I* × *v*	44,984	8,979	5.01	0.000	1.00
*I* × *t*	3,198	8,979	0.36	0.727	1.00
*O* × *v*	1,134	8,979	0.13	0.901	1.00
*O* × *t*	−4,865	8,979	−0.54	0.596	1.00
*v* × *t*	5,070	8,979	0.56	0.581	1.00

#### 3.1.1 Physical interpretation of the parameter effects.

The statistical ranking of the four process variables can be rationalised through their influence on heat input, and hence on the thermal history experienced by the inter-ring interface. Substituting the factor bounds into Eq (1) shows that deposition speed and welding current are the only two variables that alter heat input directly, and that speed does so over a wider relative range across the design space (a 1.5-fold change, versus 1.18-fold for current). This accounts for *v* returning the largest *F*-value (304.75) and *I* the second largest (188.11), while the geometric variables *O* and *t*, which modulate the thermal field only indirectly, produced *F*-values an order of magnitude lower (32.16 and 30.04).

The strongly negative quadratic coefficients for *v* (−87,489) and *I* (−68,287) indicate that fatigue life is maximised at intermediate rather than extreme settings, consistent with two competing damage mechanisms that bracket the design space. At the high-speed, low-current corner (*HI* ≈ 0.18 kJ/mm), the energy delivered per unit length is insufficient to melt the full ring wall thickness, so that the unfused region at the bore extends further and the effective load-bearing section is correspondingly reduced. Conversely, at the low-speed, high-current corner (*HI* ≈ 0.45 kJ/mm), the extended time above the austenitisation temperature and the reduced cooling rate promote epitaxial growth of coarse columnar grains oriented along the thermal gradient. Coarse structures offer fewer high-angle grain boundaries per unit crack path and therefore present weaker barriers to early crack propagation, while also lowering the resistance to crack initiation at the outer surface where the bending stress is greatest. Fatigue life is maximised where both mechanisms are simultaneously suppressed, at *HI* ≈ 0.29 kJ/mm, corresponding to the optimum reported in Section 3.3.

**Fig 11:**
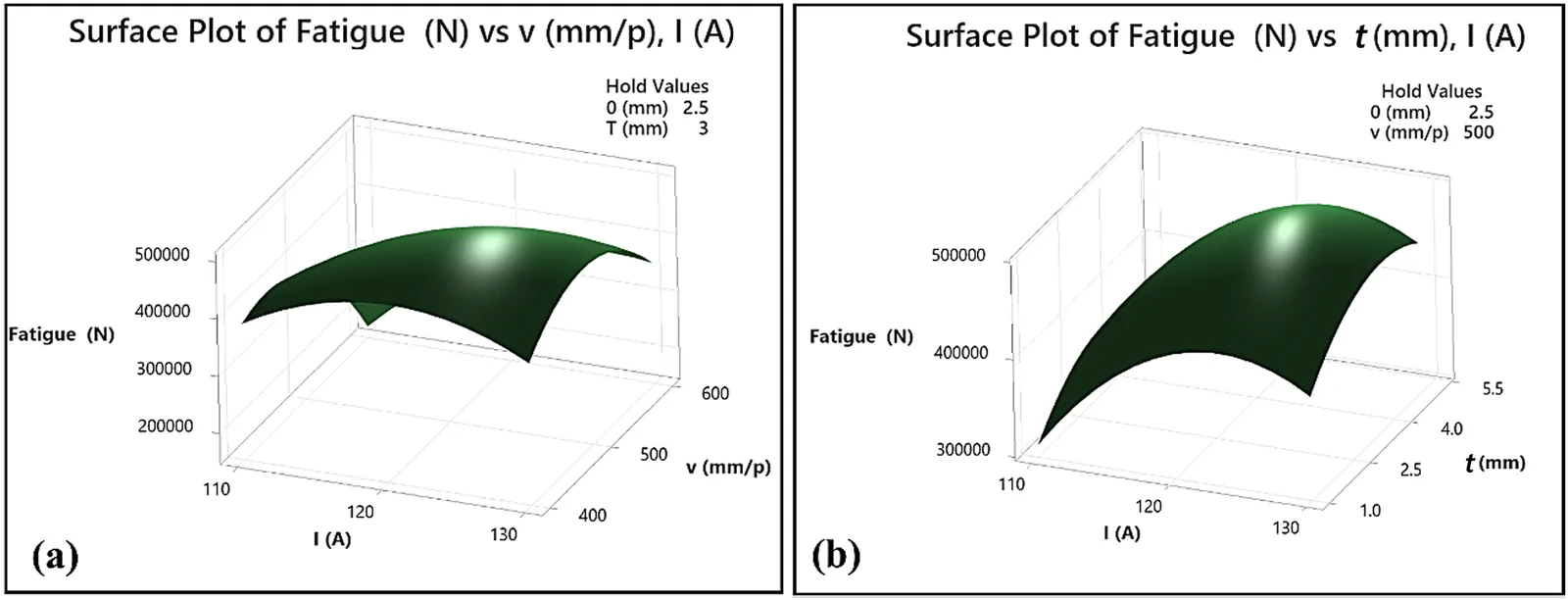
Response surface plots for fatigue life: (a) current (*I*) × deposition speed (*v*) interaction at centre levels of *O* and *t*; (b) current (*I*) × ring thickness (*t*) interaction at centre levels of *O* and *v*.

The significant *I* × *v* interaction (*F* = 25.10) follows directly from this reasoning: because current and speed appear in the numerator and denominator of the same heat-input expression, their effects are not additive. An increase in current can compensate for an increase in travel speed and vice versa, so the response surface exhibits a ridge along a line of approximately constant heat input rather than a single isolated peak (Fig 11a). The absence of significant interactions involving *O* and *t* (*p* > 0.5 in all four cases) indicates that, within the ranges investigated, bead overlap and ring thickness act through mechanisms that are essentially independent of the arc energy balance.

Bead overlap governs the extent of remelting of the previously deposited bead. The negative linear coefficient for *O* (−20,788) shows that excessive overlap is detrimental: repeated remelting of the same material increases the accumulated thermal exposure without improving fusion at the ring interface, while insufficient overlap leaves inter-bead valleys that persist as subsurface notches.

Ring thickness *t* warrants particular attention because it acts through three distinct mechanisms, all operating in the same direction. First, a thicker ring possesses greater thermal mass and conducts heat away from the deposition zone more rapidly, raising the local cooling rate and refining the solidification structure. Second, because the stack length was held constant at 120 mm, the number of ring-to-ring interfaces is inversely proportional to *t*, falling from approximately 120 interfaces at *t* = 1 mm to 24 at *t* = 5 mm — a five-fold difference across the design space. Each interface carries its own heat-affected zone, so thin rings produce a higher density of overlapping thermally affected regions and hence a less homogeneous microstructure along the specimen axis. Third, each interface constitutes a thermal contact resistance: heat crossing from one ring to the next must pass through the discrete asperity contacts of the machined mating faces, so that a larger number of interfaces impedes axial heat conduction and reduces the effectiveness of the surrounding material as a heat sink. The positive linear coefficient for *t* (+20,090) therefore reflects the combined action of increased thermal mass, reduced interface density, and reduced cumulative thermal contact resistance. The negative quadratic term (−37,028) reflects the diminishing return of these effects: beyond *t* ≈ 3.6 mm, further increases in thermal mass begin to extract heat from the interface region so rapidly that complete fusion at the ring boundary becomes progressively harder to achieve, reversing the benefit.

### 3.2 Response surface analysis

Response surface plots for the two most influential interactions *I* × *v* and *I* × *t* are presented in Fig 11. The *I* × *v* surface (Fig 11a) reveals a pronounced ridge of maximum fatigue life at intermediate to high current combined with low-to-moderate deposition speed, consistent with the negative quadratic terms for both variables. At high deposition speeds, insufficient heat input per unit length results in poor inter-layer fusion and elevated porosity, degrading fatigue performance. Conversely, excessively low speed increases heat accumulation and promotes coarser columnar grain growth, also detrimental to fatigue life.

The *I* × *t* surface (Fig 11b) demonstrates that thicker substrate rings (*t* ≈ 3.5–4 mm) combined with moderate current yield the highest fatigue lives. Thicker substrate rings act as more effective heat sinks during deposition, promoting faster solidification and finer equiaxed grain formation [42,43]. They provide greater thermal mass and superior heat dissipation, resulting in higher cooling rates near the substrate–deposit interface compared with thinner rings (*t* = 1 mm), where limited thermal mass leads to rapid heat build-up and degraded microstructural quality in the deposited layers [44,45]. This mechanism, together with the reduced interface density discussed in Section 3.1.1, is consistent with the observed effect of *t* in the response surface [46].

### 3.3 Process optimisation

Numerical optimisation using the desirability function approach identified the optimal WAAM parameter combination to maximise fatigue life within the experimental bounds. The optimal solution and corresponding model prediction are summarised in Table 6.

**Table 6:** Optimal WAAM process parameters and predicted fatigue life from the RSM model.

*I* (A)	*O* (mm)	*v* (mm/min)	*t* (mm)	Predicted *N* (cycles)
122.9	2.27	470.7	3.6	505,427 ± 10,547 (95% PI: 484,333–526,521)

The composite desirability value of 1.0 indicates that the predicted optimum lies within the feasible region and the model is well-conditioned at the optimal point. The 95% prediction interval of 484,333–526,521 cycles reflects the inherent variability associated with the WAAM process.

### 3.4 Validation and comparison

**Fig 12:**
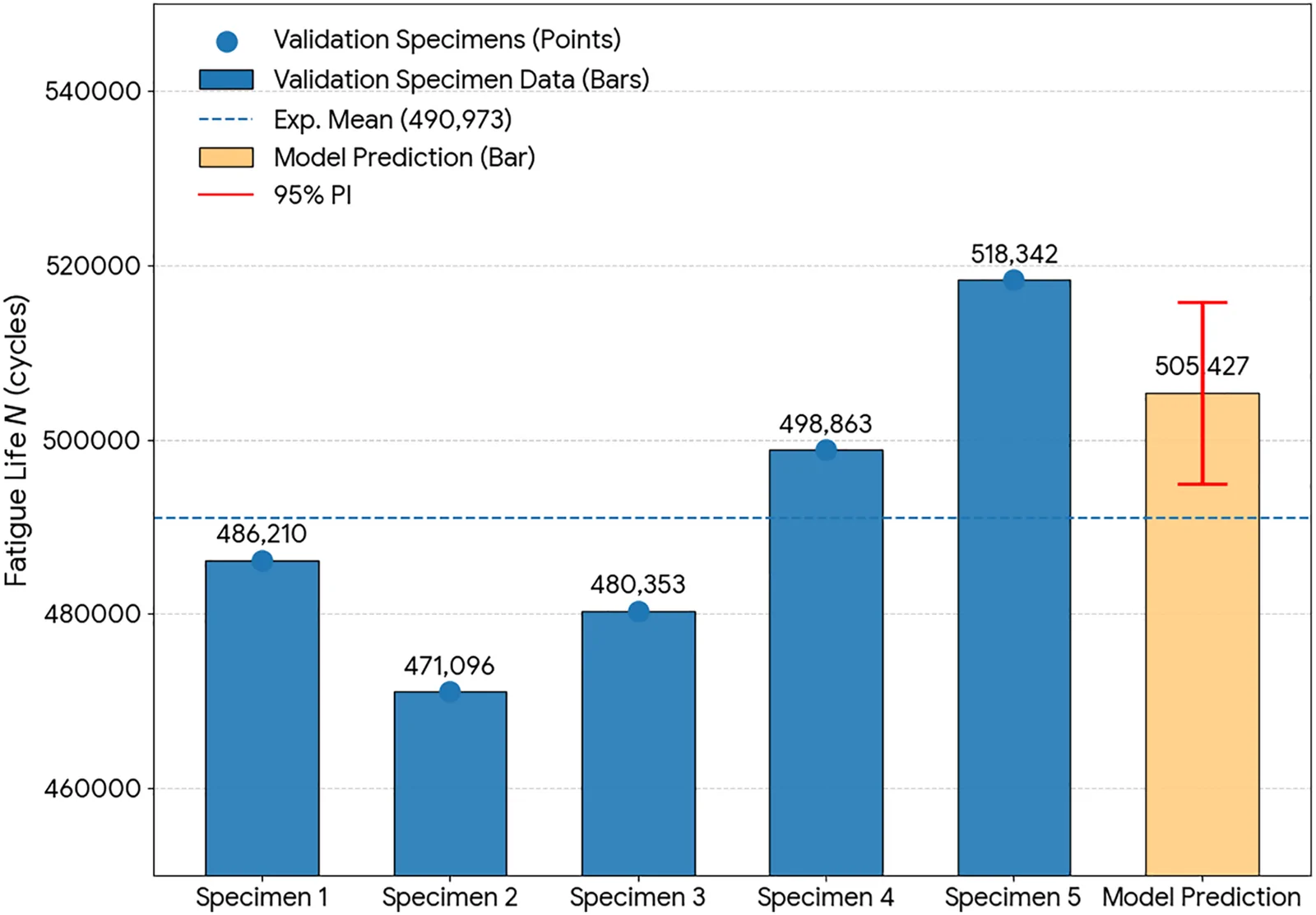
Fatigue life of five TIG-WAAM validation specimens fabricated at optimal parameters, compared with the RSM model prediction and 95% prediction interval.

**Fig 13:**
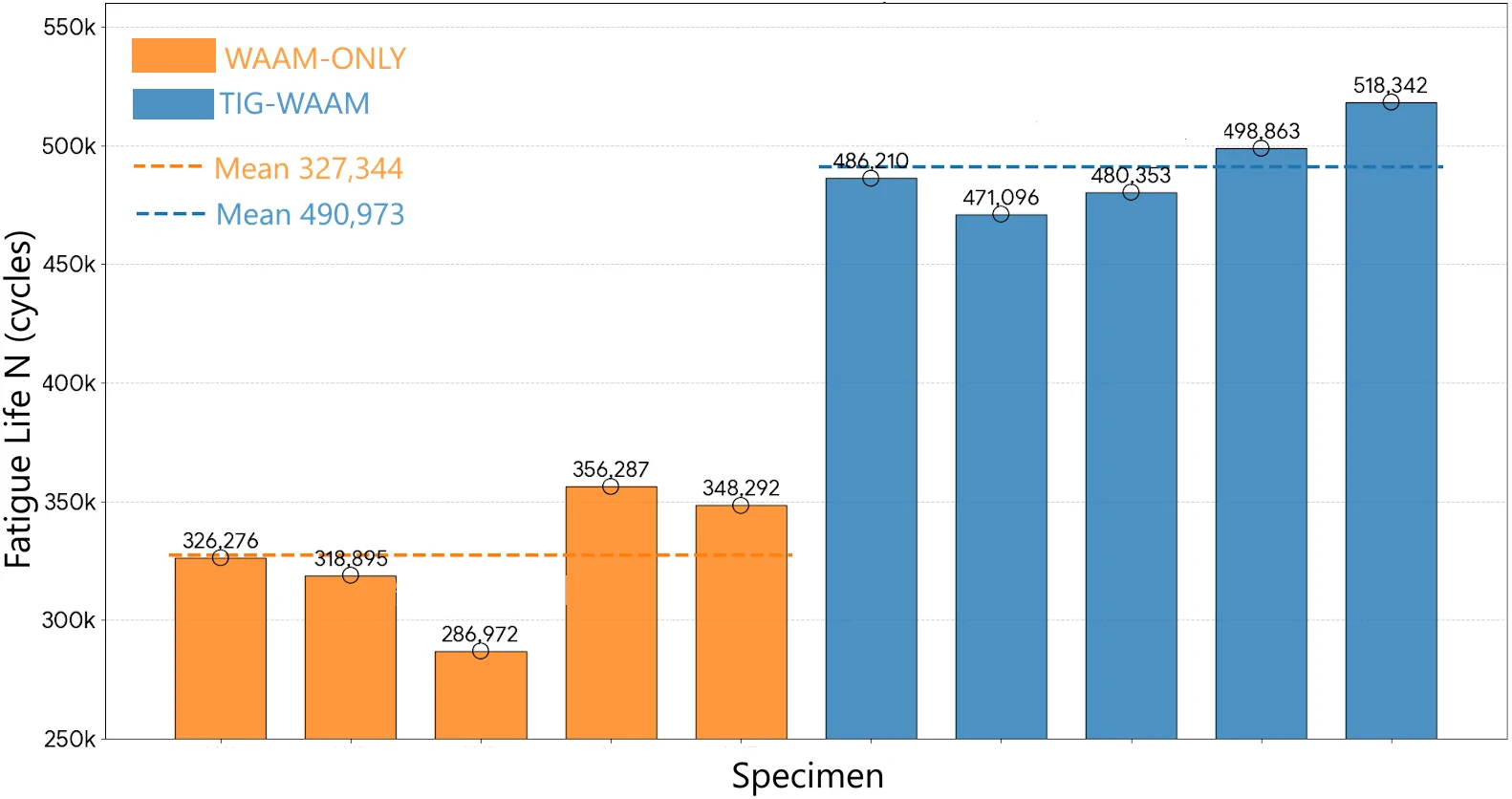
Comparison of rotating bending fatigue life between TIG-WAAM and WAAM-only specimens (*n* = 5 per group). Error bars represent one standard deviation.

Five TIG-WAAM validation specimens were fabricated at the optimal parameters (rounded to machine-settable values: *I* ≈ 123 A, *O* ≈ 2.3 mm, *v* ≈ 471 mm/min, *t* ≈ 3.6 mm) and tested under identical fatigue conditions. Five WAAM-only specimens, without TIG pre-joining, were fabricated using the same optimal WAAM parameters for direct comparison. Results are summarised in Table 7 and visualised in Fig 12 and Fig 13.

**Table 7:** Fatigue life results for validation (TIG-WAAM) and WAAM-only comparison specimens at optimal parameters.

Sample	Configuration	Fatigue Life *N* (cycles)	Mean *N* (cycles)
V1	TIG-WAAM (Optimal)	486,210; 471,096; 480,353; 498,863; 518,342	490,973
V2	WAAM-only (Optimal params, no TIG)	326,276; 318,895; 286,972; 356,287; 348,292	327,344

Fig 12 shows the fatigue life of the five TIG-WAAM validation specimens compared with the RSM model prediction and its 95% prediction interval. The TIG-WAAM validation specimens achieved a mean fatigue life of 490,973 cycles (individual results: 486,210; 471,096; 480,353; 498,863; 518,342 cycles), which is only 2.86% below the RSM model prediction of 505,427 cycles and lies within the 95% prediction interval of the model (484,333–526,521 cycles). The small negative deviation is not unexpected given the inherent stochastic nature of the WAAM process, which introduces several sources of variability that are not fully captured by the deterministic RSM model. These include: (i) arc stability fluctuations arising from power source dynamics and contact tip wear during multi-layer deposition; (ii) minor surface irregularities on the substrate ring outer diameter after concentric grinding, which can locally alter thermal contact and heat dissipation at the deposition interface; and (iii) sub-surface micro-defects such as micro-porosity and localised lack-of-fusion, which are stochastic in nature and cannot be eliminated solely through process parameter optimisation. Such process-inherent variability is consistent with the coefficient of variation of 3.7% observed among the five validation specimens and has been similarly reported in fatigue studies of other WAAM low-carbon steel components [26–28]. The 2.86% deviation between the mean validation result and the model prediction is considered acceptable for engineering purposes and confirms the practical utility of the developed response surface model.

Fig 13 presents the comparison of rotating bending fatigue life between TIG-WAAM and WAAM-only specimens (*n* = 5 per group). The WAAM-only specimens yielded a mean fatigue life of 327,344 cycles, corresponding to an improvement of approximately 50% conferred by TIG pre-joining. The coefficient of variation for TIG-WAAM specimens was 3.7%, compared with 8.3% for WAAM-only specimens, indicating that TIG pre-joining also substantially improves repeatability.

**Fig 14:**
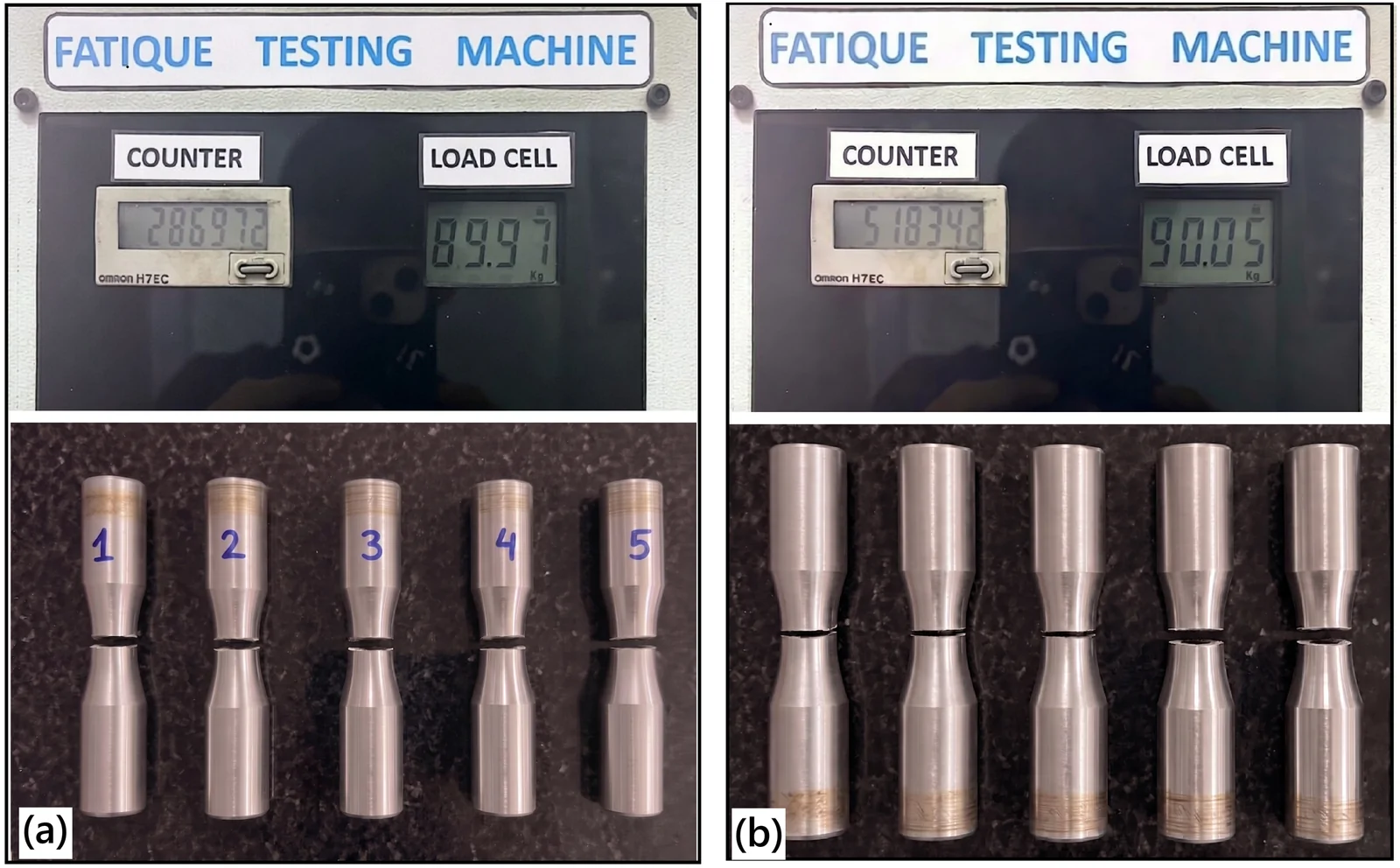
Complete set of ten specimens after rotating bending fatigue testing and fractured specimens tested at optimal WAAM parameters.

Representative fractured specimens, together with the fatigue testing machine displays, are shown in Fig 14. Fig 14a presents the WAAM-only specimen with the lowest recorded fatigue life (286,972 cycles), while Fig 14b presents TIG-WAAM specimen with the highest (518,342 cycles).

### 3.5 Reliability and scatter analysis

**Fig 15:**
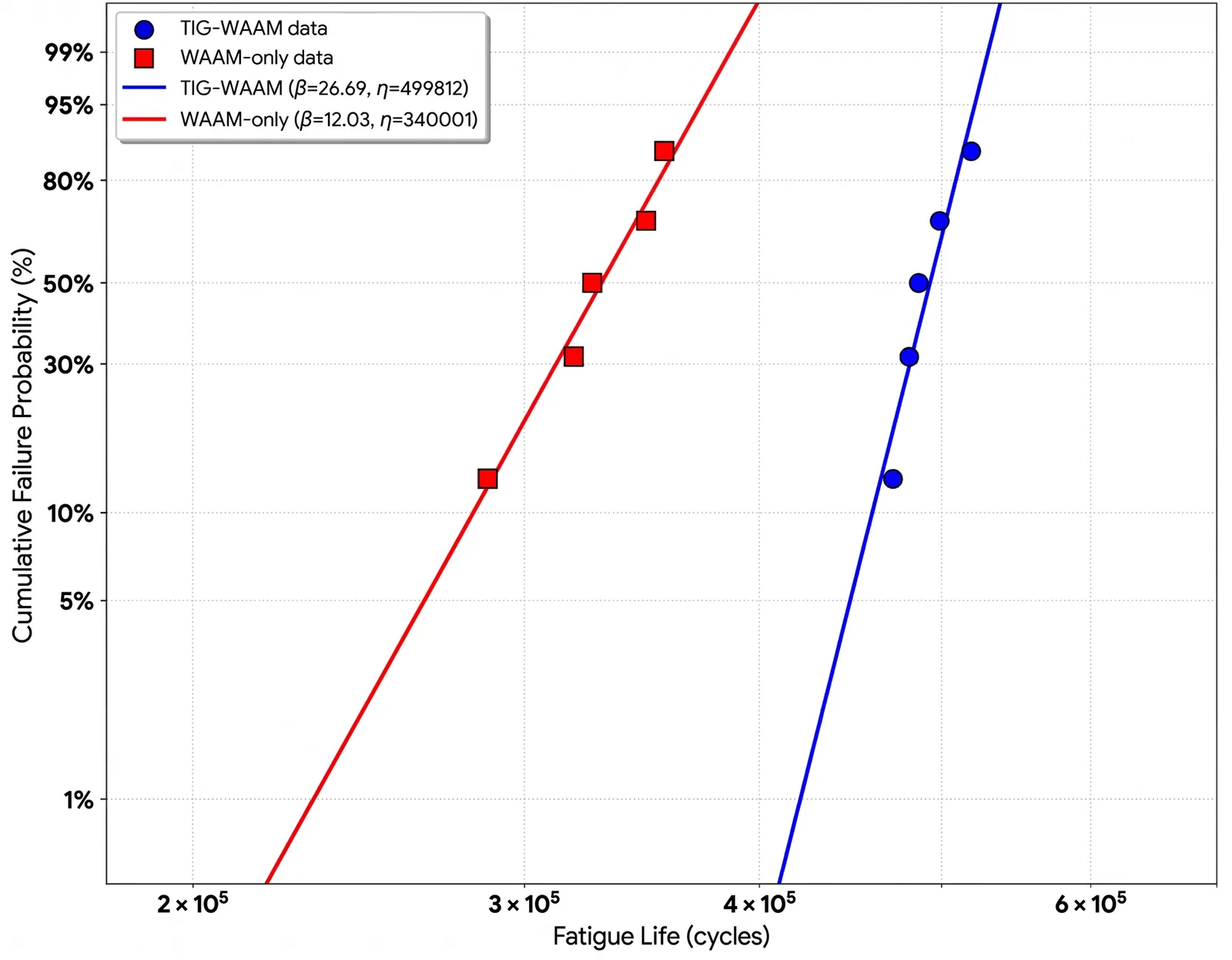
Weibull probability plot of rotating bending fatigue life for TIG-WAAM (*β* = 26.69, *η* = 499,812 cycles) and WAAM-only (*β* = 12.03, *η* = 340,001 cycles) validation specimens (*n* = 5 per group).

A two-parameter Weibull analysis was performed on the fatigue life data from both validation groups to characterise the reliability and scatter behaviour of each configuration. The fitted parameters and derived characteristic lives are summarised in Table 8, and the Weibull probability plot is presented in Fig 15.

**Table 8:** Weibull parameters and characteristic lives for the two validation configurations (*n* = 5 per group).

Configuration	Shape *β*	Scale *η* (cycles)	B10 (cycles)	B50 (cycles)	CV (%)
TIG-WAAM	26.69	499,812	459,395	492,995	3.7
WAAM-only	12.03	340,001	282,007	329,801	8.3
Improvement	—	+47.0%	+62.9%	+49.5%	—

The shape parameter for the TIG-WAAM group is more than twice that of the WAAM-only group, indicating substantially lower dispersion in fatigue life, and this is corroborated by the directly computed coefficients of variation (3.7% versus 8.3%), which involve no distributional assumption. The B10 characteristic life — the life at 10% cumulative failure probability, widely used as a conservative design threshold — was 459,395 cycles for TIG-WAAM specimens against 282,007 cycles for WAAM-only specimens, a 62.9% improvement at this reliability level. It is notable that the improvement at B10 exceeds the improvement in mean life, which reflects the fact that TIG pre-joining benefits the lower tail of the distribution disproportionately: the mechanism it eliminates is the same one that produces the poorest individual results. The B50 median life estimates are consistent with the arithmetic means reported in Table 7, confirming the internal coherence of the dataset.

Two caveats apply. First, least-squares estimation of the Weibull shape parameter from five specimens is subject to substantial positive bias, so the absolute value of *β* = 26.69 should not be interpreted as a reliable measure of scatter in its own right; the comparison between groups, both estimated by an identical procedure from equal sample sizes, is the informative quantity. Second, no confidence bounds are placed on the fitted parameters, as these would be uninformatively wide at this sample size. Expanded testing with *n* ≥ 10 per group and formal confidence bound analysis are recommended in Section 4.5.

As an independent check, a two-sample Welch’s *t*-test (corrected for unequal variances) yielded *t*(7.92) = 11.13, *p* < 0.001. The difference in mean fatigue life was 163,629 cycles, with an approximate 95% confidence interval of 129,800–197,400 cycles; expressed as a ratio, the point estimate corresponds to a 50% increase in mean life with the confidence interval spanning approximately 40%–60%. The magnitude of the improvement is therefore established with reasonable confidence, whereas its precise value is not, and the figure of 50% should be read as a central estimate for this specific material system and parameter set rather than as a transferable constant. Given the small sample size, normality cannot be formally verified and statistical power remains limited; the result should be interpreted as corroborative rather than definitive.

## 4. Discussion

### 4.1 Microstructural analysis

#### 4.1.1 Grain structure.

**Fig 16:**
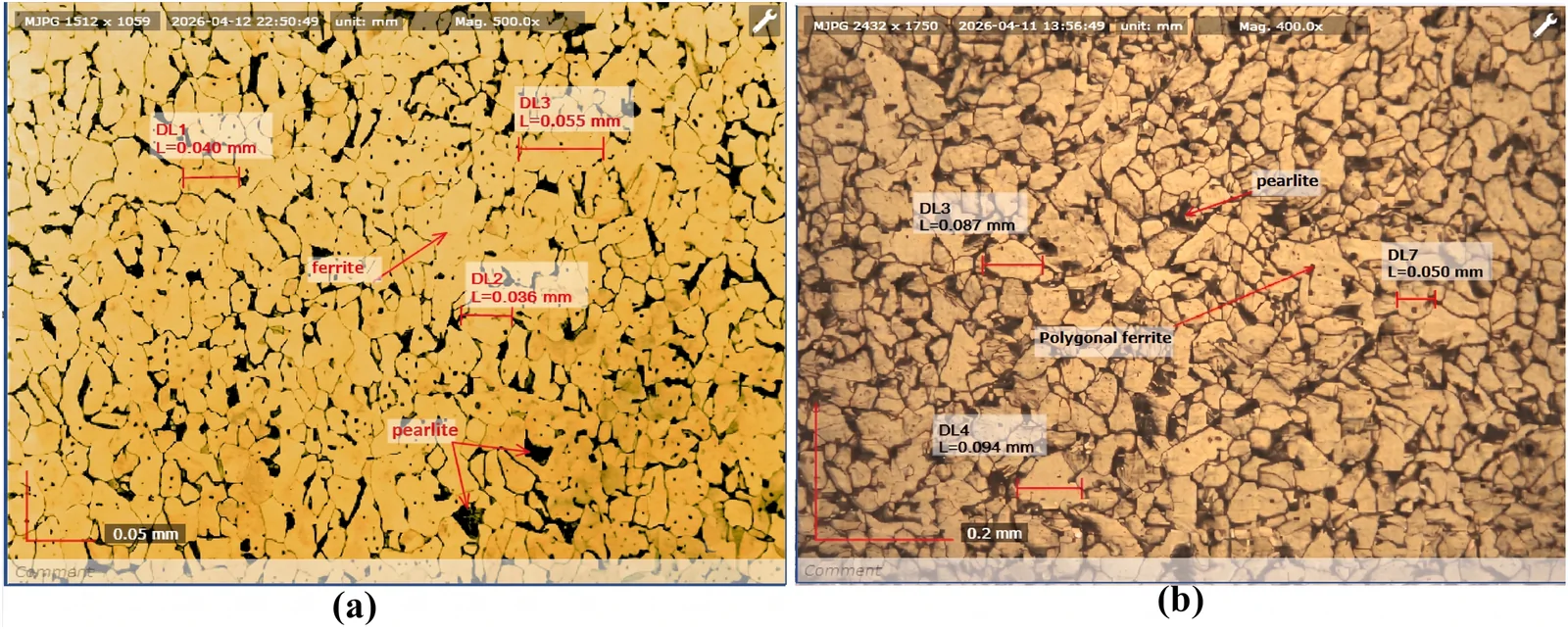
Optical micrographs of the fusion zone: (a) TIG-WAAM specimen at 500 × showing refined polygonal ferrite with pearlite at grain boundaries, and (b) WAAM-only specimen at 400 × showing a coarser and less uniform grain structure. Individual grain diameter measurements are annotated for illustration; label numbering is assigned automatically by the microscope software and is not sequential.

Optical micrographs of the fusion zone for both specimen types are presented in Fig 16. In TIG-WAAM specimens, the interface region between the deposited WAAM bead and the substrate exhibits a refined grain size of approximately 35–55 µm, consisting of uniformly distributed polygonal ferrite and pearlite (Fig 16a). This finer microstructure is attributed to the additional thermal cycle imposed by WAAM deposition on the pre-existing TIG HAZ. The heat from WAAM deposition reheats the TIG HAZ to temperatures within the intercritical (Ac1–Ac3) and subcritical ranges, inducing partial recrystallisation and grain refinement through the formation of fine ferrite nuclei at prior austenite grain boundaries. This phenomenon, analogous to HAZ refinement observed in multi-pass welding, results in a more homogeneous grain structure that resists fatigue crack initiation and short-crack propagation [13,14,47].

In contrast, WAAM-only specimens show a coarser grain structure with grain diameters ranging from approximately 50–94 µm at the fusion zone, where WAAM material is deposited directly onto the unmodified substrate rings (Fig 16b). Beyond the shift towards coarser grains, the WAAM-only condition exhibits a spread more than twice as wide (44 µm versus 20 µm), indicating a markedly less uniform microstructure — a characteristic that parallels the higher scatter in fatigue life recorded for the same configuration. The deposited zone in WAAM-only specimens additionally exhibits a coarser columnar grain structure, which further reduces resistance to crack propagation as grain boundaries aligned perpendicular to the stress axis facilitate Stage II fatigue crack growth [16].

#### 4.1.2 Interfacial fusion quality.

To assess the quality of the metallurgical bond at the macroscopic scale, longitudinal sections were prepared through the gauge region of failed specimens by wire electrical discharge machining along the axial direction, followed by standard metallographic polishing and optical microscopy under identical etching conditions for both configurations.

**Fig 17:**
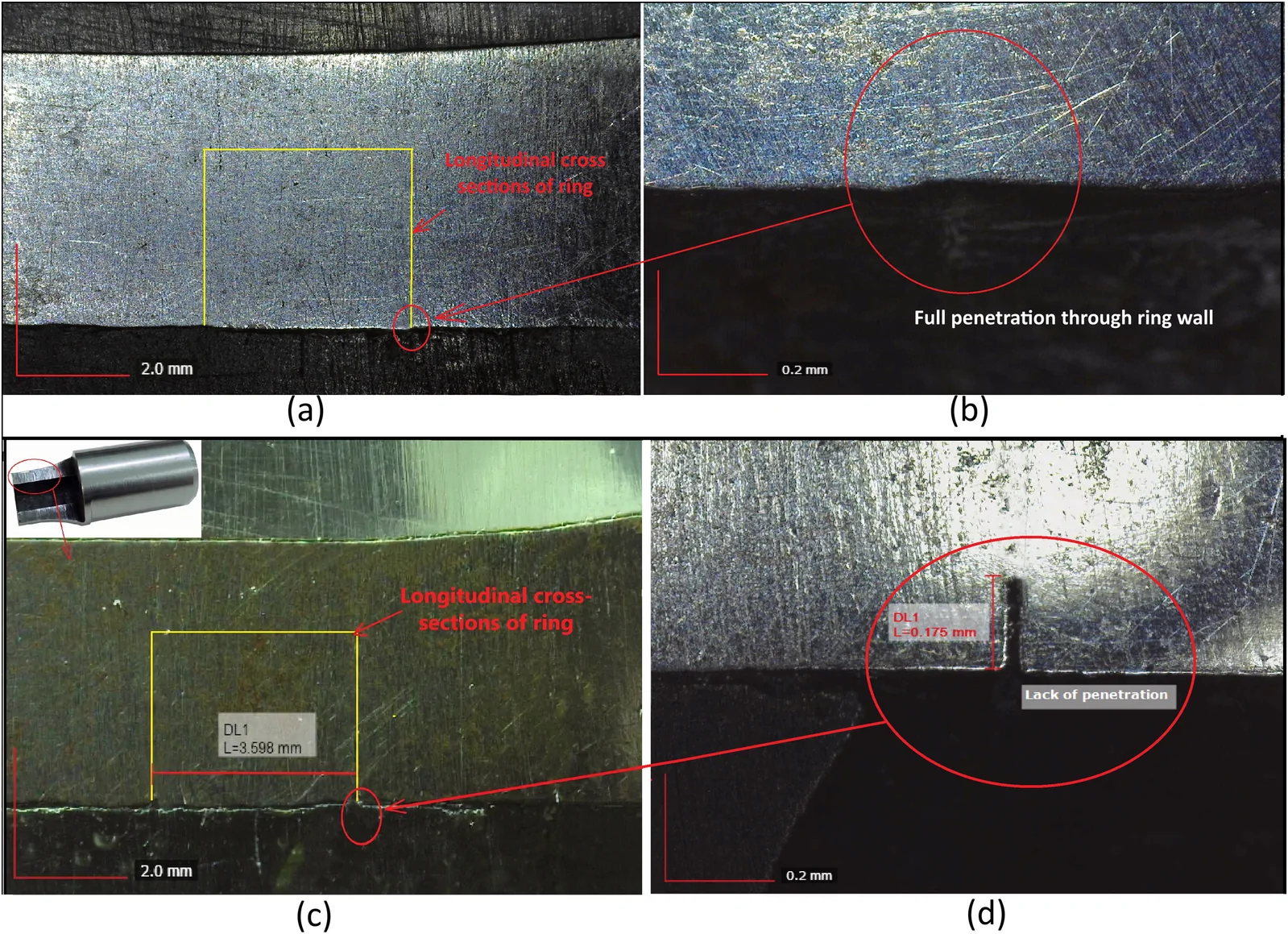
Longitudinal EDM cross-sections through the gauge region, shown as an overview and an enlarged detail for each configuration. (a, b) TIG-WAAM specimen: the enlarged view shows continuous metal to the bore surface with no resolvable discontinuity, indicating full penetration through the ring wall. (c, d) WAAM-only specimen: at the equivalent location the enlarged view reveals incomplete root penetration extending 0.175 mm from the bore surface. All images were acquired at identical magnifications (55× and 250×) under identical surface preparation and illumination conditions.

In the WAAM-only specimens, each ring-to-ring interface exhibited an unfused region extending 0.175 mm from the bore surface (Fig 17d). Because deposition proceeds from the outer surface inward, the root of the interface at the bore is the last region reached by the molten pool, and the observed value corresponds to complete fusion across approximately 91% of the 2 mm ring wall with the remaining 9% at the root left unbonded. This condition, incomplete root penetration in the terminology of ISO 6520−1, was typical of the interfaces examined rather than isolated to a single location, and its extent varied around the circumference of any given interface rather than being uniform.

In the TIG pre-joined specimens, no interfacial feature was resolvable at any of the interface positions (Fig 17a, 17b). Fusion extended through the full ring wall thickness, and in some locations a small volume of metal had penetrated slightly into the bore, confirming that the molten pool had traversed the complete wall section. The comparison is therefore not one of degree but of kind: 0.175 mm of unbonded root at every interface in the WAAM-only condition, against complete continuity in the TIG pre-joined condition.

The mechanical consequence of the unfused annulus is a reduction in the effective load-bearing section. Treating the unbonded region as an increase in effective bore diameter from 10.00 mm to 10.35 mm reduces the section modulus from 518 mm^3^ to 510 mm^3^, a loss of 1.5%, and correspondingly raises the nominal outer-fibre stress amplitude by approximately 1.6%. Adopting a Basquin exponent representative of ferritic–pearlitic steel in the high-cycle regime, a stress increase of this magnitude would account for a life reduction of the order of 15%, against the 33% reduction actually observed between the two configurations. Even allowing for the uncertainty in the exponent, which was not determined in this study, the geometric effect is insufficient to explain the majority of the difference. The remainder must be attributed to the condition of the material at the crack initiation site, which is examined in Section 4.2. It should be noted that this estimate is based on a representative value of the unfused depth measured on a single sectioned specimen and is intended as an order-of-magnitude assessment rather than a precise partition.

A further consequence follows from the circumferential non-uniformity of the unfused region. Because its depth varies around the bore, and because the specimen rotates during testing, the effective section modulus presented to the bending load is not constant with angular position, and its distribution differs from specimen to specimen. This provides a direct mechanistic explanation for the higher scatter recorded for the WAAM-only group (CV = 8.3%) compared with the highly repeatable TIG-WAAM results (CV = 3.7%), and for the disproportionate improvement in B10 life reported in Section 3.5.

#### 4.1.3 Microhardness distribution.

**Fig 18:**
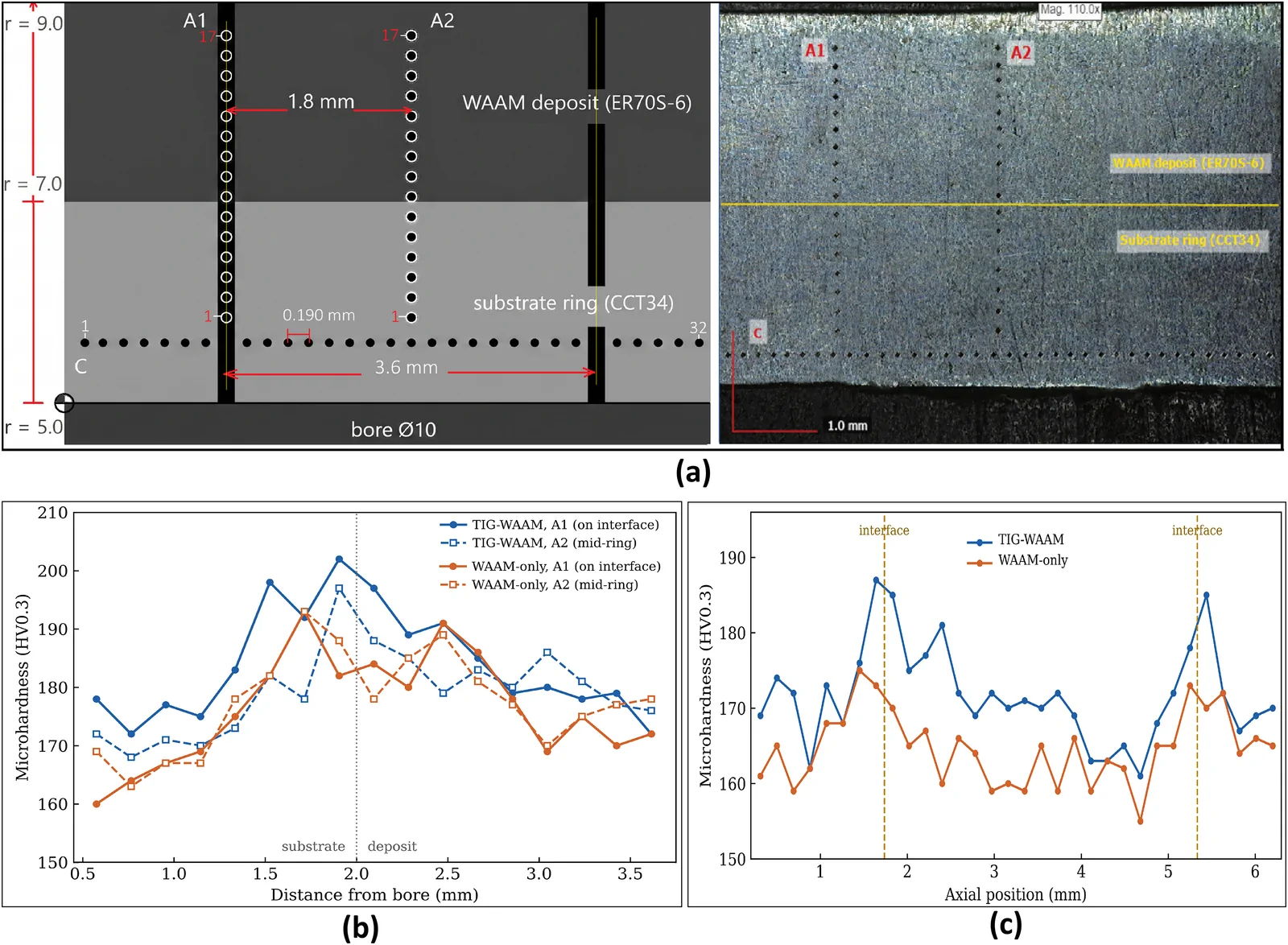
Vickers microhardness measurements. (a) Traverse layout on the longitudinal section: schematic (left) and the corresponding indentation array on the TIG-WAAM specimen at 135× (right); traverse A1 lies on a ring-to-ring interface, A2 at the mid-ring position 1.8 mm away, and C runs axially at *r* = 5.345 mm across two consecutive interfaces. The ring interfaces, shown as vertical lines in the schematic, are not optically resolvable in the TIG-WAAM specimen owing to complete fusion; their positions were established from the fracture plane and the known ring thickness. (b) Radial profiles along A1 and A2 for both configurations, with the substrate–deposit boundary at *r* = 7.0 mm indicated. (c) Axial profiles along C, with the ring interface positions indicated.

The measured microhardness profiles are presented in Fig 18 and summarised in Table 9. The two radial traverses were positioned to isolate the effect of the TIG pass: A1 lies on a ring-to-ring interface and A2 at the mid-ring position 1.8 mm away, on the same polished section, at the same radii, and prepared and measured under identical conditions. Any difference between them within a given specimen is therefore attributable to axial position relative to the interface rather than to specimen preparation, instrument calibration, or bulk material variation.

**Table 9:** Vickers microhardness (HV0.3) by region and traverse. Values are mean ± standard deviation of individual indentations.

Region	Traverse	WAAM-only	TIG-WAAM	Difference
Substrate ring (*r* = 5.58–6.91 mm)	A1, on interface	174.0 ± 10.4	184.6 ± 10.6	+10.6 (*p* = 0.004)
	A2, mid-ring	175.9 ± 10.3	176.4 ± 8.9	+0.5 (*p* = 0.85)
WAAM deposit (*r* = 7.10–8.62 mm)	A1	178.3 ± 7.2	183.3 ± 7.3	+5.0 (*p* = 0.03)
	A2	178.9 ± 5.2	181.7 ± 3.9	+2.8 (*p* = 0.29)
Near bore (*r* = 5.345 mm)	C, whole traverse	164.7 ± 4.7	171.8 ± 6.3	+7.1 (*p* < 0.001)

Within the substrate ring the resulting comparison forms a consistent pattern. At the mid-ring position the two configurations are indistinguishable (176.4 versus 175.9 HV0.3, *p* = 0.85), and within the WAAM-only specimen there is no elevation at the interface relative to the mid-ring position (−1.9 HV0.3, *p* = 0.21). The TIG pre-joined specimen behaves differently in both respects: its interface traverse is 8.2 HV0.3 harder than its own mid-ring traverse (*p* = 0.001) and 10.6 HV0.3 harder than the corresponding interface traverse in the WAAM-only specimen (*p* = 0.004), reaching a local maximum of 202 HV0.3. The two null comparisons and the two positive comparisons together localise the effect: the TIG pass produces a measurable hardened zone confined to the interface region, and the substrate ring is otherwise unaffected by it.

Within the WAAM deposit the picture is different. The four traverses converge to a narrow band between 178 and 183 HV0.3, and the difference between configurations is small and not consistently significant. At the outermost measured radii, where fatigue cracks initiated in every specimen, the two configurations differ by approximately 5 HV0.3. This is an important observation for the interpretation of the fatigue results. Vickers hardness reflects resistance to plastic indentation and correlates principally with yield strength; the two deposits are therefore comparable in bulk mechanical properties at the site where cracks initiate. The difference in fatigue behaviour cannot be attributed to one deposit simply being stronger than the other. It must instead arise from the microstructural feature that does differ, namely the grain size and its distribution documented in Section 4.1.1, which governs slip band formation and crack nucleation but which, by the Hall–Petch relation, would account for only 8–10 HV0.3 across the observed range and is therefore not resolvable against the scatter of these measurements.

The axial traverse near the bore separates two independent effects. Both configurations exhibit a local hardness maximum at each ring interface, of closely similar magnitude relative to the mid-ring baseline (+8.9 HV0.3 in the WAAM-only specimen, *p* < 0.001; + 8.8 HV0.3 in the TIG-WAAM specimen, *p* = 0.002). Because this elevation is present in the specimen that received no TIG pass, it cannot be attributed to TIG pre-joining, and is more plausibly associated with the machined condition of the ring mating faces or with the local thermal history adjacent to the joint plane. Superimposed on this is a uniform offset: the TIG pre-joined specimen is approximately 8.5 HV0.3 harder than the WAAM-only specimen at every axial position, both at the interfaces (+8.4 HV0.3, *p* = 0.005) and away from them (+8.6 HV0.3, *p* < 0.001). This uniform elevation of the near-bore material is consistent with the TIG pass having melted through the full ring wall and thermally processed the region adjacent to the bore, whereas in the WAAM-only configuration this region remained unfused and thermally unaffected — the same conclusion reached independently from the metallographic sections in Section 4.1.2.

A further observation concerns the absolute values. The substrate ring measured 176 HV0.3 away from the interface in both configurations, corresponding by the customary approximation *σ*_b_ ≈ 3.3 HV to a tensile strength of approximately 580 MPa. This exceeds the specified range for CCT34 in the as-delivered condition (340–450 MPa, TCVN 1765−75 [53]). Since the elevation is common to both configurations, it is attributable to the WAAM deposition itself: the ring wall is only 2 mm thick and is subjected to repeated thermal cycling by successive deposited layers, producing grain refinement and, at the cooling rates involved, transformation products harder than the ferrite–pearlite structure of the delivered material. The substrate ring in the finished component is therefore not in its as-delivered metallurgical condition, and the bending stresses reported in Section 2.5 correspond to a considerably smaller fraction of the local yield strength than the nominal specification would suggest.

### 4.2 Fractographic analysis

#### 4.2.1 Crack initiation sites.

In both configurations, fatigue cracks initiated at the outer surface of the gauge section, where the bending stress reaches its maximum. No initiation was observed at the bore, and none at the unfused root regions identified in Section 4.1.2. This is consistent with a stress-based assessment: at the bore radius the bending stress amplitude is 116 MPa, compared with 208.5 MPa at the outer fibre, and the stress intensity factor range associated with an unfused depth of 0.175 mm at that location is approximately 2–3 MPa·m^0.5^, well below the threshold for fatigue crack propagation in ferritic–pearlitic steel. The unfused root therefore does not act as a propagating crack, and its influence on fatigue life is exerted through the section-loss and non-uniformity mechanisms described in Section 4.1.2 rather than by providing an initiation site.

**Fig 19:**
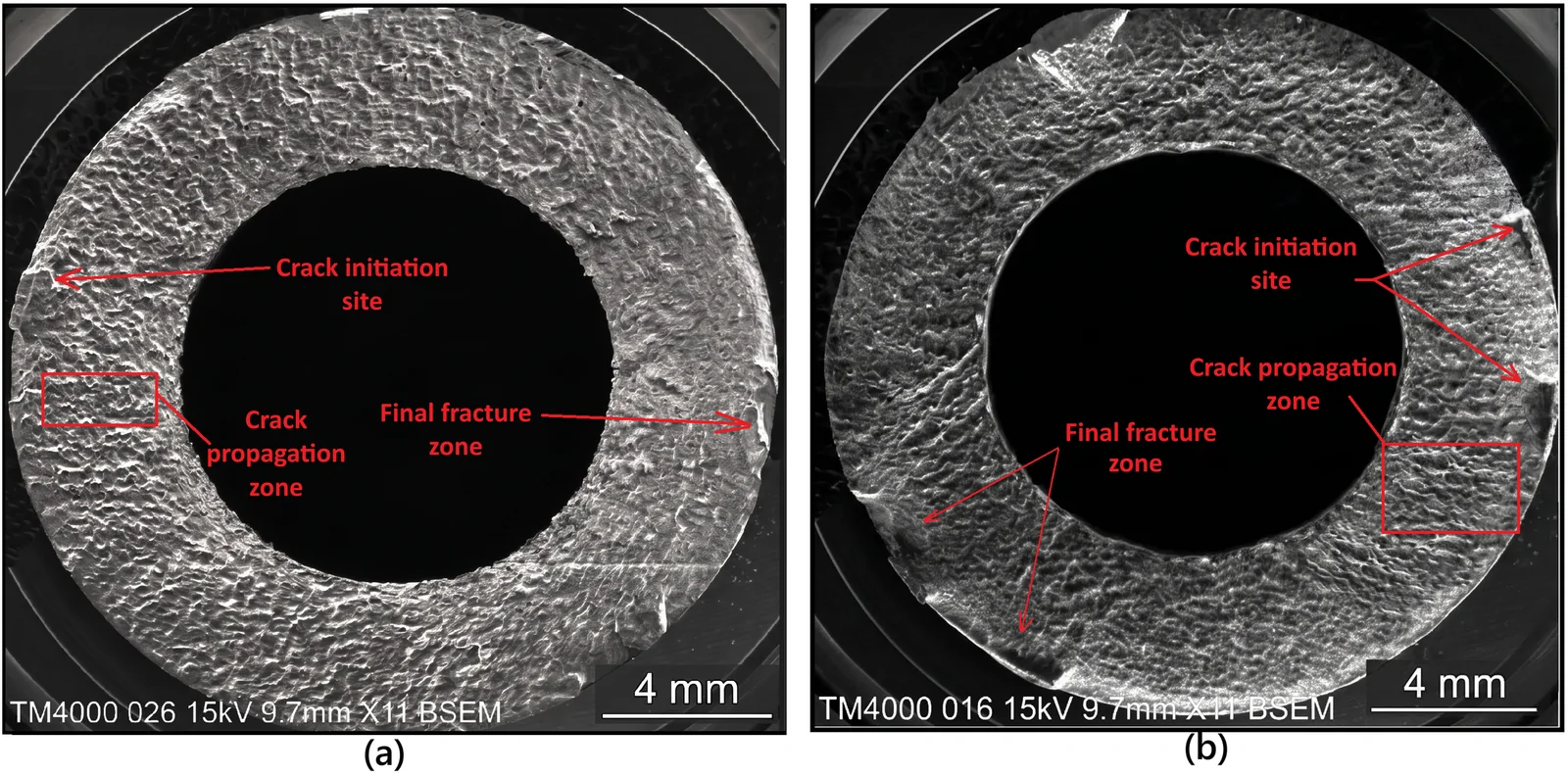
Macro-scale SEM fractographs of the fatigue fracture surfaces at 11 × : (a) TIG-WAAM specimen showing a single crack initiation site at the outer periphery, the adjacent crack propagation zone, and the final fracture zone located approximately diametrically opposite; (b) WAAM-only specimen showing two independent initiation sites on the outer periphery separated by approximately 90° of arc, with the corresponding propagation zone and final fracture zone.

The two configurations differ markedly in the number of initiation sites. The TIG-WAAM specimen exhibits a single initiation region, visible as a smooth, flattened crescent at the outer periphery (Fig 19a), from which the crack front advanced as a single coherent boundary across the section. The WAAM-only specimen, by contrast, exhibits two distinct initiation regions on the outer periphery, separated by approximately 90° of arc (Fig 19b). These developed independently before their crack fronts met, and the resulting coalescence produced the irregular fracture topography evident in the propagation region.

Because both configurations were machined and polished to the same surface finish, and because initiation occurred at the surface in both cases, the difference in initiation behaviour cannot be attributed to surface condition. Nor can it be attributed to bulk strength, which the microhardness measurements of Section 4.1.3 show to be comparable at this location. It is instead consistent with the microstructural difference documented in Section 4.1.1. Crack initiation at a free surface under high-cycle fatigue is governed by the formation of persistent slip bands, and the probability of a given surface region reaching the initiation condition depends on local grain size and orientation. In a microstructure with a narrow grain size distribution, initiation is dominated by the small number of most favourably oriented grains, producing a single site. In a microstructure with a distribution more than twice as wide, a larger population of grains lies close to the critical condition, so that several sites reach initiation within a comparable number of cycles. The multi-site initiation observed in the WAAM-only specimens is therefore a direct expression of the greater microstructural heterogeneity of that condition, and not merely a coincidental feature.

#### 4.2.2 Crack propagation region.

The propagation region is identifiable in both specimens as a band of comparatively smooth, flattened texture adjacent to the initiation sites, produced by repeated contact between the crack faces during the fully reversed loading cycle.

At higher magnification, the propagation region of the WAAM-only specimen exhibits a stepped morphology comprising large, differently inclined facets separated by distinct ridges, together with networks of secondary micro-cracks (Fig 21b). The facet dimensions, of the order of 100 µm, are comparable to the prior grain size measured in this configuration (50–94 µm), indicating that the crack path was strongly influenced by the coarse grain structure, with the crack front repeatedly deflected at grain boundaries and propagating along preferred crystallographic planes within individual grains. Such deflection is characteristic of crack growth through a coarse, heterogeneous microstructure. The secondary micro-cracking observed alongside the main fracture path indicates that the advancing front interacted repeatedly with local discontinuities, each interaction producing a local perturbation of the crack front geometry.

The coalescence of the two independent crack fronts in the WAAM-only specimen produced an additional feature: because the two fronts propagated on slightly different planes, their junction generated a step in the fracture surface that persisted through the remainder of the propagation region. No equivalent feature is present in the TIG-WAAM specimen, where the single crack front advanced on a continuous plane.

The transition between the fatigue propagation region and the final overload region could not be delineated with sufficient confidence at the magnification employed for the relative extent of each region to be quantified. The character of crack growth was therefore assessed from higher-magnification examination of the region adjacent to the initiation sites rather than from areal measurement.

#### 4.2.3 Final fracture zone.

The final fracture zone is distinguished from the propagation region by an abrupt change in surface texture, from the smooth, flattened fatigue region to a rough, fibrous morphology. In both configurations this zone is located approximately diametrically opposite the initiation site, consistent with a crack front that advanced circumferentially in both directions from the origin before the remaining ligament failed by overload.

**Fig 20:**
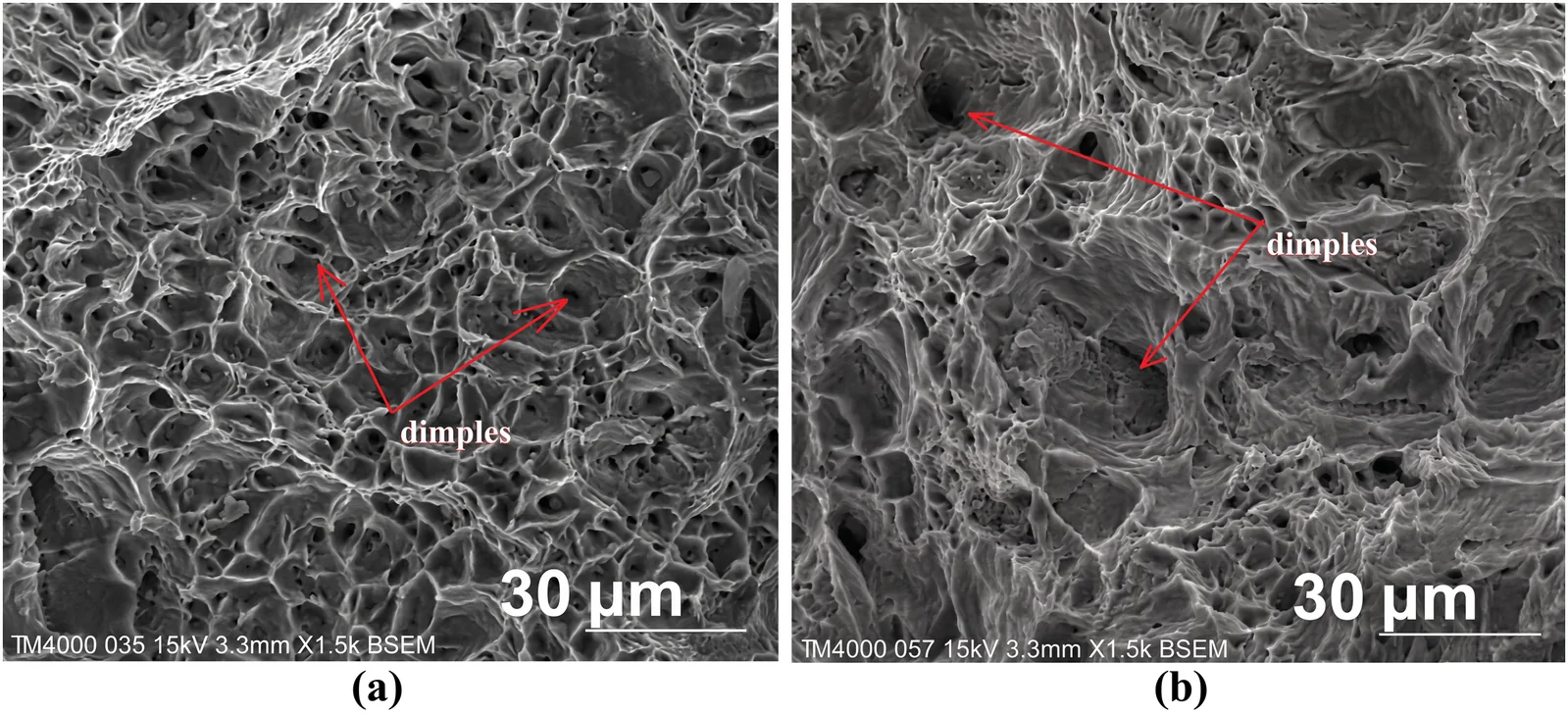
High-magnification SEM fractographs of the final overload zone at 1500 × : (a) fine, uniformly sized dimples in the TIG-WAAM specimen, and (b) coarser, heterogeneously distributed dimples in the WAAM-only specimen.

**Fig 21:**
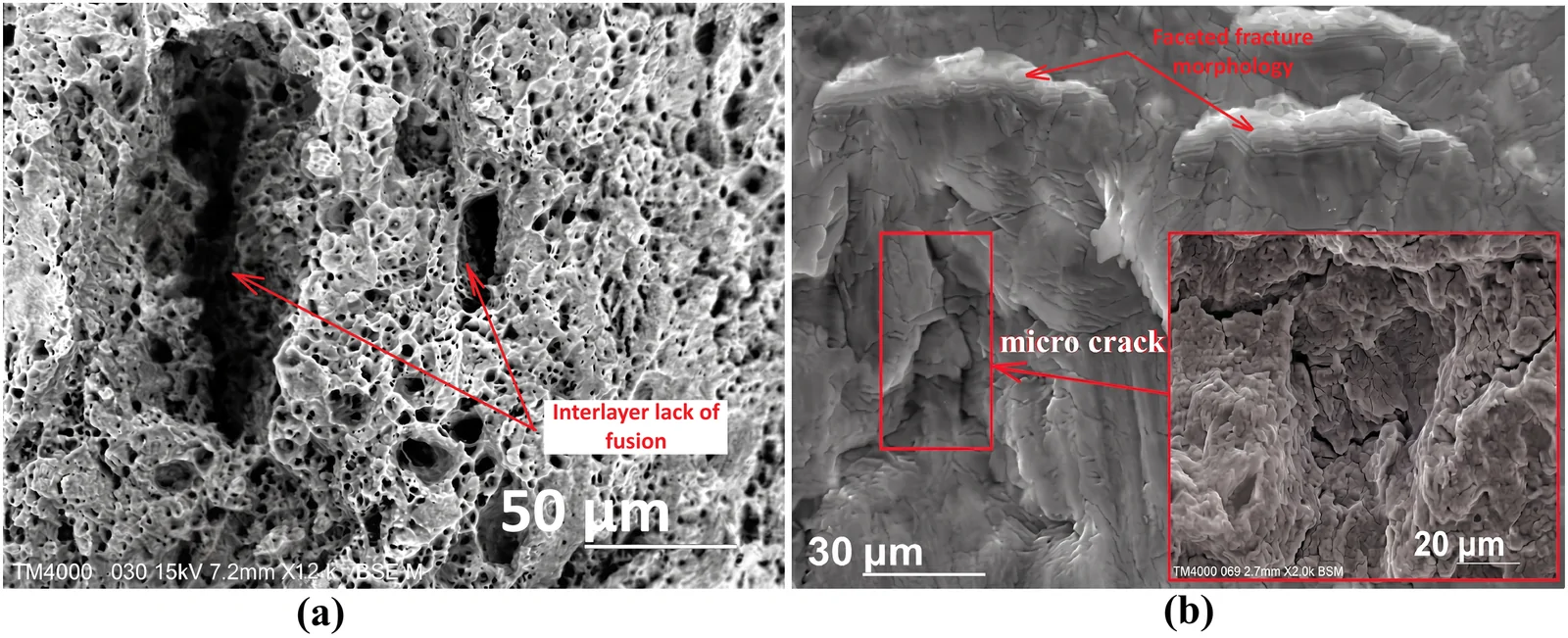
High-magnification SEM fractographs of the WAAM-only specimen: (a) final fracture zone at 1.2k× showing coarse dimples together with elongated voids attributable to interlayer lack of fusion within the deposit; (b) propagation region showing a faceted fracture morphology with facet dimensions comparable to the prior grain size, and networks of secondary micro-cracks (inset, 2.0k×).

High-magnification examination of the overload zone reveals differences in local ductility and void evolution (Fig 20). The TIG-WAAM specimen shows a fine ductile fracture morphology characterised by the nucleation of numerous small, uniformly sized micro-voids (Fig 20a), reflecting a refined microstructure with a high density of void nucleation sites and efficient energy absorption distributed across a large number of small voids during final rupture. The WAAM-only specimen exhibits larger and more heterogeneously distributed dimples (Fig 20b). It is important to note that the larger dimple size in this context does not indicate superior ductility; rather, it reflects void growth dominated by a smaller number of preferential nucleation sites within a coarser grain structure, together with pre-existing micro-defects such as micro-porosity and interlayer lack of fusion, such that individual voids grow to a greater size before coalescence. Measured dimple diameters were 10–20 µm for TIG-WAAM specimens compared with 20–40 µm for WAAM-only specimens. Elongated voids attributable to interlayer lack of fusion within the deposit are visible in the final fracture zone of the WAAM-only specimen (Fig 21a); these are distinct in origin from the incomplete root penetration at the ring interfaces described in Section 4.1.2.

Taken together, the three fracture regions describe a consistent sequence. The refined and more uniform microstructure of the TIG pre-joined condition delays crack initiation and confines it to a single site; it then presents a finer structure to the advancing crack, producing a more regular propagation path; and it finally sustains a larger fatigue crack before overload, in part because the load-bearing section is not reduced by an unfused root. The improvement in fatigue life is therefore not attributable to any single stage but to a cumulative advantage across all three.

### 4.3 Mechanism of fatigue life enhancement by TIG pre-joining

The improvement in mean rotating bending fatigue life conferred by TIG pre-joining arises from three mechanisms operating at different stages of the fatigue process.

The first governs crack initiation and is microstructural. In both configurations, cracks initiated at the machined outer surface rather than at any interfacial feature, so the initiation life is determined by the local resistance of the material to persistent slip band formation. Microhardness measurements show that the two deposits are comparable in bulk strength at this location (Section 4.1.3), so the difference cannot be attributed to one deposit being stronger than the other. It arises instead from the grain structure: the refined polygonal ferrite produced in the TIG pre-joined condition (35–55 µm) raises the resistance to slip band formation relative to the coarser structure of the WAAM-only condition (50–94 µm), and, more significantly, the grain size distribution is less than half as wide, which restricts the population of grains capable of initiating a crack and results in the single-site initiation observed fractographically. The origin of this refinement is the additional thermal cycle imposed by WAAM deposition on the pre-existing TIG heat-affected zone: reheating into the intercritical (Ac1–Ac3) and subcritical ranges induces partial recrystallisation and the nucleation of fine ferrite at prior austenite grain boundaries, a phenomenon analogous to heat-affected zone refinement in multi-pass welding [13,14,47].

The second governs the propagation and final fracture stages and is geometric. The unfused root region present at every ring interface in the WAAM-only condition reduces the effective section modulus by approximately 1.5% and raises the nominal outer-fibre stress by approximately 1.6%, which alone accounts for a life reduction of the order of 15%. The unfused region also reduces the ligament available to sustain the load once a fatigue crack has developed, advancing the transition to unstable fracture. TIG pre-joining eliminates this loss entirely by achieving complete penetration through the full ring wall, as confirmed both metallographically (Section 4.1.2) and by the elevated near-bore hardness of the TIG pre-joined condition (Section 4.1.3).

The third governs the reproducibility of the result. The depth of the unfused root varies around the circumference of each interface and differs between nominally identical specimens, so that the effective section presented to the rotating bending load is neither constant with angular position nor consistent from specimen to specimen. This is the direct source of the elevated scatter in the WAAM-only group (CV = 8.3% against 3.7%) and explains why the improvement at the B10 reliability level (62.9%) exceeds the improvement in mean life: the feature eliminated by TIG pre-joining is the same one responsible for the poorest individual outcomes.

The multi-scale evidence supports this account. Longitudinal sections establish the presence and extent of the unfused root and its absence after pre-joining (Fig 17); optical microscopy establishes the difference in grain size and its distribution (Fig 16); microhardness profiling localises the metallurgical effect of the TIG pass to the interface region and confirms comparable bulk properties in the deposit (Fig 18); and fractography establishes the difference in initiation multiplicity, propagation path, and overload behaviour (Figs 19–21). No single observation is decisive in isolation, but the three mechanisms are mutually consistent and together account for both the higher mean fatigue life and the markedly improved reproducibility.

Direct quantitative comparison of fatigue life with existing WAAM literature is not straightforward, as no prior study has investigated rotating bending fatigue of hollow shaft geometries fabricated on stacked substrate ring preforms with a TIG pre-joining step. Studies such as Ermakova et al. [28], which reported uniaxial fatigue data for solid WAAM ER70S-6 bar specimens, and Fang et al. [26], which examined high-cycle fatigue of WAAM carbon steel plates, employed fundamentally different specimen geometries, loading modes, and fabrication configurations that preclude direct numerical comparison. Nevertheless, the present results are broadly consistent with the range of fatigue performance reported for WAAM ER70S-6 low-carbon steel under comparable stress amplitudes and heat input conditions [26–28]. The absence of directly comparable literature data further underscores the novelty of the present work and highlights the need for standardised fatigue testing protocols for WAAM components with complex substrate geometries.

### 4.4 Engineering implications

The combination of RSM-optimised WAAM deposition and TIG pre-joining provides a practical, low-cost manufacturing route for hollow shafts with integrated internal cooling channels. The identified optimal process window (*I* ≈ 123 A, *O* ≈ 2.3 mm, *v* ≈ 471 mm/min, *t* ≈ 3.6 mm) can be implemented directly on existing 4-axis CNC-WAAM systems without requiring additional equipment. The 2.9% margin between the mean validation fatigue life (490,973 cycles) and the RSM prediction (505,427 cycles) indicates slightly conservative model behaviour that is advantageous for engineering safety margins.

**Fig 22:**
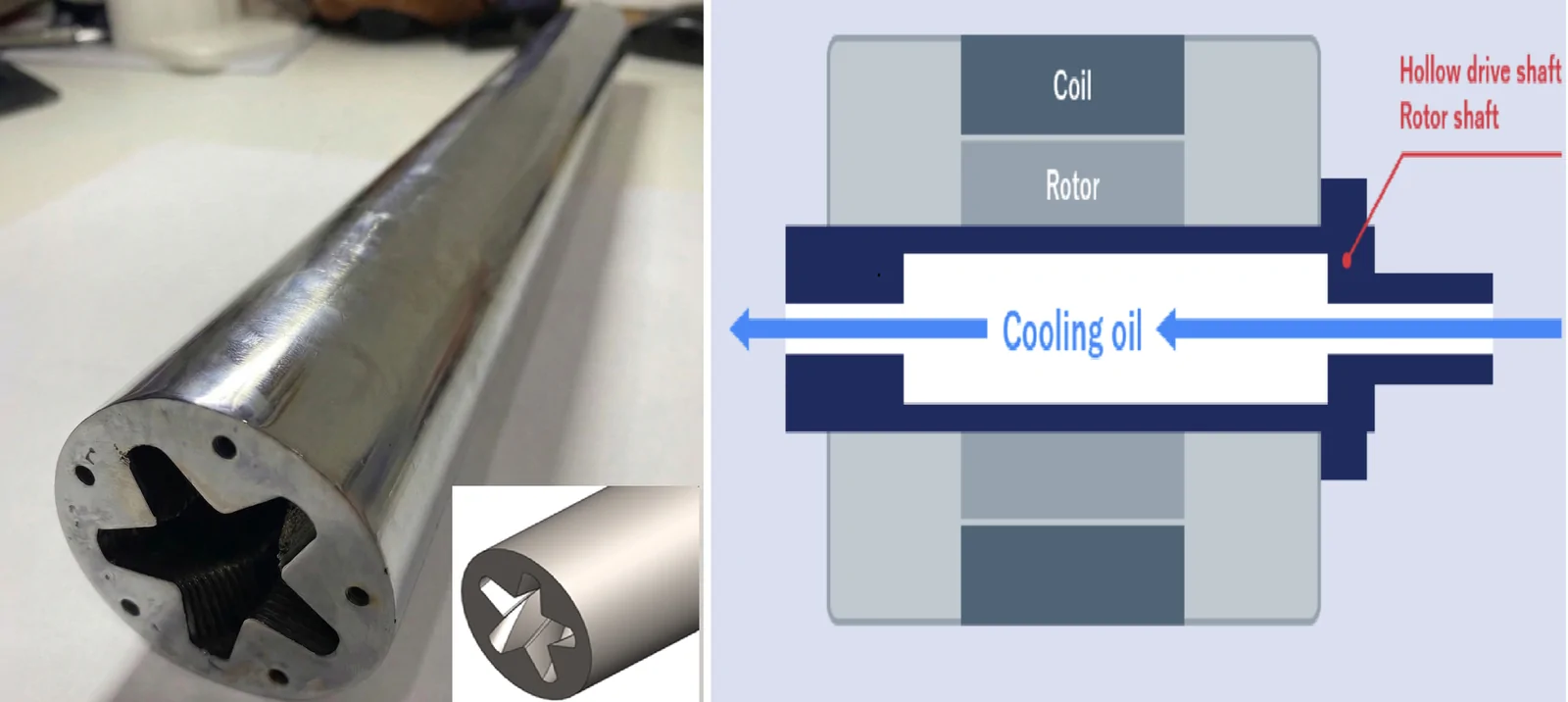
Preliminary prototype of a hollow drive shaft with internal spiral cooling channel fabricated using the TIG-WAAM process: (a) as-machined component showing the star-shaped internal cross-section, and (b) schematic illustration of the integrated internal spiral cooling channel.

To demonstrate the versatility of the developed TIG-WAAM approach for more complex internal geometries, a preliminary prototype of a hollow drive shaft with an internal spiral cooling channel was fabricated using the optimised parameters on stacked substrate rings. The prototype, after finish machining, exhibits a continuous internal helical cooling channel designed for efficient heat dissipation in high-performance rotor applications (Fig 22). This demonstration confirms that the method can be extended beyond simple cylindrical bores to non-circular and helical internal features while maintaining excellent interfacial bonding.

Although the parameter optimisation and TIG-WAAM forming method developed in this work represent only the initial step toward practical industrial application, achieving the highest quality and longest service life in real components will require the integration of appropriate post-processing techniques, including heat treatment and advanced surface treatments, to meet the most stringent performance requirements [48]. These post-processing strategies, together with full-scale fatigue validation on complex geometries such as the prototype presented here, constitute key directions for future research.

### 4.5 Limitations and future work

While the findings of this study provide evidence for the fatigue life enhancement achievable through TIG pre-joining combined with RSM-optimised WAAM deposition, several limitations should be acknowledged. First, although the validation comparison was conducted with *n* = 5 specimens per group, further expansion of the sample size (*n* ≥ 10 per group) would enable more precise estimation and tighter confidence bounds on the Weibull parameters. Second, all fatigue tests were conducted at a single stress amplitude (*σ*_a_ = 208.5 MPa), and a complete S–N curve spanning multiple stress levels was not constructed. Generating full S–N data across a range of stress amplitudes would allow determination of the endurance limit and facilitate component-level fatigue life prediction under variable amplitude loading. Third, the present work was limited to a single material system (ER70S-6 wire on CCT34 substrate); the generalisability of TIG pre-joining benefits to other wire–substrate combinations such as stainless steel or aluminium alloys remains to be investigated.

The microhardness characterisation carries its own limitations. Each traverse was measured once, without replication, so that the reported dispersion reflects local microstructural variation rather than measurement repeatability, and the two specimens examined represent the extremes of their respective validation groups rather than the group means. The paired statistical comparisons along each traverse assume independence between successive indentation pairs; spatial autocorrelation along the traverse is likely, so the reported *p*-values should be read as supporting indicators rather than as strict inferential evidence.

Beyond these considerations, four further aspects fall outside the scope of the present work but are directly relevant to practical deployment. First, residual stresses were not measured. Although both TIG pre-joining and WAAM deposition were followed by stress-relief annealing according to ISO 17663 [40], the residual stress state actually remaining at the interface was not quantified, and since residual stress superimposes directly on the applied cyclic stress in rotating bending, its measurement by X-ray diffraction or the hole-drilling method would allow the mechanical and metallurgical contributions to the observed improvement to be separated. Second, the influence of post-processing treatments was not examined; shot peening, deep rolling, or low-plasticity burnishing introduce compressive residual stress at the surface and could plausibly act in combination with, rather than in place of, the interfacial consolidation achieved by TIG pre-joining — a possibility of particular interest given that crack initiation in this study occurred at the outer surface in both configurations. Third, all testing was performed in ambient laboratory air, whereas hollow shafts carrying internal coolant are exposed to a corrosive medium at the bore surface; corrosion-fatigue evaluation in a representative coolant environment is therefore necessary before service application, particularly as the ring interfaces intersect the bore and the unfused root regions identified in the WAAM-only condition would present preferential paths for environmental attack. Fourth, loading was restricted to fully reversed uniaxial bending, while shafts in service experience combined bending and torsion; multiaxial fatigue testing under proportional and non-proportional loading would establish whether the benefit of TIG pre-joining persists when the interfaces are subjected to a shear-dominated stress state, which is a more demanding condition for a transverse bonded plane. Complementary characterisation by electron backscatter diffraction and X-ray diffraction, to resolve grain orientation, boundary character distribution, and phase fractions across the interface, is also identified as a priority.

A further observation is recorded here as a limitation because it was not instrumented. A loosening of the clamping rod was noted on WAAM-only assemblies following deposition, which was not apparent for TIG pre-joined assemblies. This suggests that differential thermal expansion during deposition may permit relative micro-movement at the ring interfaces when the stack is held only by mechanical clamping, whereas TIG pre-joining renders the stack a rigid body. Such movement beneath the molten pool could plausibly contribute to the incomplete root penetration observed in the WAAM-only configuration. This mechanism was not measured in the present study and is offered as an observation warranting dedicated investigation, for example by in-situ measurement of clamping force or interface displacement during deposition.

## 5. Conclusions

This study conducted a systematic investigation using RSM-CCD to evaluate the effects of WAAM process parameters on the rotating bending fatigue life of hollow shafts fabricated on TIG pre-joined substrate rings, followed by a direct comparison with WAAM-only specimens. The following conclusions can be drawn:

1. The second-order response surface model developed from 30 experimental runs demonstrated excellent predictive capability, with *R*² = 98.20%, *R*²_adj_ = 96.52%, and *R*²_pred_ = 90.72%. The non-significant lack-of-fit (*p* = 0.113) confirmed that the model adequately represented the experimental data.
2. Deposition speed (*v*) and welding current (*I*) were identified as the most significant parameters influencing fatigue life, with *F*-values of 304.75 and 188.11 respectively. The interaction between current and deposition speed (*I* × *v*) was the only statistically significant two-way interaction (*F* = 25.10, *p* < 0.001). The effect of ring thickness is attributable to three concurrent mechanisms: increased thermal mass, reduced interface density along the specimen axis, and reduced cumulative thermal contact resistance.
3. The optimal WAAM parameters were determined as *I* ≈ 123 A, *O* ≈ 2.3 mm, *v* ≈ 471 mm/min, and *t* ≈ 3.6 mm. The RSM model predicted a maximum fatigue life of 505,427 cycles at these parameters. Validation tests (*n* = 5) achieved a mean fatigue life of 490,973 cycles, which lies within the 95% prediction interval of the model.
4. TIG pre-joining improved the mean rotating bending fatigue life by approximately 50% compared with WAAM-only specimens (490,973 cycles versus 327,344 cycles; 95% confidence interval on the difference spanning approximately 40%–60%) and reduced the variability in fatigue life (coefficient of variation of 3.7% versus 8.3%). At the B10 reliability level the improvement was 62.9%.
5. Metallographic sectioning showed that WAAM deposition alone left an unfused region extending 0.175 mm from the bore at each ring-to-ring interface, corresponding to incomplete root penetration across approximately 9% of the ring wall, whereas TIG pre-joining achieved complete fusion through the full wall thickness. Optical microscopy revealed a refined polygonal ferrite structure (35–55 µm) in TIG-WAAM specimens against a coarser and more than twice as widely distributed structure (50–94 µm) in WAAM-only specimens. Microhardness profiling localised the metallurgical effect of the TIG pass to the interface region (+10.6 HV0.3, *p* = 0.004) while showing the two configurations to be indistinguishable away from the interface (*p* = 0.85). Fractography showed single-site crack initiation, a regular propagation path, and fine uniform dimples in the TIG-WAAM condition, against two independent initiation sites, a faceted propagation morphology reflecting the coarse grain structure, and larger heterogeneous dimples in the WAAM-only condition.
6. The combined strategy of TIG pre-joining and optimised WAAM parameters provides a practical, cost-effective manufacturing route for producing hollow shafts with complex internal geometries and significantly enhanced fatigue performance.
7. The improvement reported here was established at a single stress amplitude with five specimens per configuration, under uniaxial rotating bending, on machined surfaces, and in ambient air. Its extension to full S–N characterisation, to as-deposited surface conditions, to multiaxial loading, and to service environments involving internal coolant remains to be verified, and the residual stress state at the consolidated interface has not yet been quantified.

## Supporting information

S1 File Experimental data support. (ZIP)

S1 File Structure observision. (ZIP)
