# Supplementary material for: Comparative study of the rotating bending fatigue performance of wire arc additively manufactured hollow shafts with and without TIG pre-joining
Source: PLoS One. 2026 Sep 18;21(9):e0357916. doi: 10.1371/journal.pone.0357916 (PMC13588359; doi:10.1371/journal.pone.0357916)
Supplement: Supplementary file 1 [file pone.0357916.s004.docx]

# Response to Editor

**Manuscript ID:** PONE-D-26-20584

**Original title:** Comparative Study on Rotating Bending Fatigue Performance of Wire Arc Additively Manufactured Hollow Shafts: Effect of TIG Pre-Joining

**Revised title:** Comparative Study of the Rotating Bending Fatigue Performance of Wire Arc Additively Manufactured Hollow Shafts with and without TIG Pre-Joining

Dear Dr. Arora,

We thank you for the constructive and detailed evaluation of our manuscript. The comments have led to substantial improvements, particularly in the framing of the study’s novelty, the justification of the material system, the depth of the mechanistic discussion, and the rigour with which our statistical claims are expressed.

Two additions deserve mention at the outset. First, following Reviewer #1’s recommendation, we have carried out a new microhardness characterisation campaign. This was designed as a controlled comparison traverses positioned on a ring-to-ring interface and at the mid-ring position on the same section and it has produced what we regard as the most decisive single piece of evidence in the revised manuscript. Second, we verified every numerical value in the manuscript during revision. This identified several internal inconsistencies that were not flagged by the reviewers; all have been corrected and are itemised at the end of this letter for transparency.

We should also report that this exercise obliged us to revise our own interpretation of the failure mechanism. The original manuscript attributed the reduced fatigue life of WAAM-only specimens to interfacial defects acting as crack initiation sites. Closer examination of the fracture surfaces established that cracks initiated at the outer surface in both configurations, and that the unfused region lies at the bore where the bending stress is lowest. The revised mechanism, presented in Section 4.3, is correspondingly different and, we believe, better supported.

All changes are marked in the tracked-changes version. Reviewer comments are reproduced in **bold**; our responses follow.

## Response to the Academic Editor

### Journal Requirement 1 — PLOS ONE formatting

The manuscript has been reformatted according to the PLOS ONE template. All tables are now sequentially numbered with captions placed above (the automatic numbering fields in the original submission had failed, producing captions without numbers); all five equations are numbered; figures are cited as “Fig 1” throughout; and figure files have been prepared separately in the required format.

### Journal Requirements 2 and 3 — Funding Information and Financial Disclosure

This work was supported by Ho Chi Minh City University of Technology and Engineering under grant number T2026-245, awarded to the corresponding author. The Financial Disclosure statement reads: “This research was funded by Ho Chi Minh City University of Technology and Engineering under grant number T2026-245, awarded to Van-Minh Nguyen. The funder had no role in study design, data collection and analysis, decision to publish, or preparation of the manuscript.” The grant number has been entered in the online Funding Information form. In accordance with PLOS requirements, funding information does not appear in the manuscript file; the Acknowledgments section refers only to the provision of laboratory facilities.

### Journal Requirement 4 — Recommended citations

We are grateful for the guidance that recommended works should be evaluated for relevance rather than cited automatically. We have applied that standard and report the outcome under Comments 1.4 and 1.9 below.

## Response to Reviewer #1

We are grateful to Reviewer #1 for a demanding and detailed assessment. The points raised regarding novelty, material justification, statistical inference, microstructural characterisation, and fractographic depth have materially strengthened the manuscript.

### Comment 1.1 — Novelty relative to existing work on WAAM fatigue improvement

**The novelty of incorporating TIG pre-joining prior to WAAM deposition should be more clearly established against existing studies on WAAM fatigue enhancement techniques.**

We accept that the original framing did not make the distinction clear, and we have rewritten the closing portion of the Introduction accordingly.

The revised text makes the following argument. Existing strategies for improving WAAM fatigue performance post-deposition heat treatment, mechanical surface treatments such as shot peening or machining, and in-process interventions such as interpass rolling all operate on a monolithic deposit built on a simple flat or bar substrate. The fatigue-critical features they address are consequently internal to the deposit: porosity, columnar grain morphology, or as-built surface waviness.

The configuration studied here is different in kind. Building a hollow shaft on a stack of discrete substrate rings introduces a fatigue-critical feature absent from monolithic WAAM entirely: a series of transverse ring-to-ring interfaces distributed along the component axis. Because deposition proceeds from the outer surface inward, the root of each interface lies at the bore and is the last region reached by the molten pool, so that any incompleteness in fusion is concentrated precisely where the process has least control. Neither post-deposition heat treatment nor surface treatment can remedy such a discontinuity, because it lies beneath the deposit and is created before deposition begins. It can only be eliminated at the assembly stage, which is the specific function of the TIG pre-joining step.

We have also strengthened the survey of prior work to demonstrate that this configuration is not covered by existing literature. Ermakova et al. [28] characterised uniaxial and multiaxial fatigue of WAAM ER70S-6 but tested solid bar specimens from a monolithic wall; Fang et al. [26] examined high-cycle fatigue of WAAM carbon steel plates. In neither case did the specimen contain a substrate interface. Work on hollow and tubular WAAM geometries has concentrated on deposition strategy and dimensional accuracy rather than cyclic performance, and studies of WAAM on structured substrates have reported static properties without extending to fatigue [29,30].

We would add that framing the novelty in terms of the geometry and substrate architecture, rather than in terms of TIG welding as such, is in our view the accurate description of the contribution, and we thank the Reviewer for prompting us to state it precisely.

### Comment 1.2 — Justification for the choice of ER70S-6 wire and CCT34 substrate rings

**The manuscript lacks sufficient justification for selecting ER70S-6 wire and CCT34 low-carbon steel substrate rings.**

A dedicated justification has been added to Section 2.1, together with a new table of nominal chemical compositions (Table 1).

The rationale rests on three grounds. Metallurgically, the bond between the deposited bead and the substrate ring is formed by arc melting and solidification and is therefore, in essence, a fusion weld; its integrity is governed by the same factors that control weldability in conventional arc welding of low-carbon steels, namely compatibility of composition, carbon equivalent, and solidification range. ER70S-6 and CCT34 are both low-carbon, low-alloy steels with closely comparable carbon and manganese contents, so dilution at the interface produces a fusion zone spanning two similar parent compositions rather than a dissimilar-metal gap. This avoids the hard martensitic transition layers, sharp hardness discontinuities, and differential thermal contraction associated with dissimilar-alloy combinations, all of which are known promoters of interfacial crack initiation under cyclic loading. The microhardness profiles now reported in Section 4.1.3 provide direct experimental confirmation: no sharp hardness discontinuity was observed at the substrate–deposit boundary in either configuration.

Functionally, the elevated silicon and manganese content of ER70S-6 provides strong deoxidation, which matters when depositing onto a ground ring surface that may retain a thin oxide film, and the wire exhibits stable bead formation across a wide current range a prerequisite for the broad parameter window explored here.

Practically, ER70S-6 in 1.2 mm diameter is the most widely available and lowest-cost solid welding wire in the Vietnamese market and is compatible with standard MIG power sources, while CCT34 seamless tube is a commodity product available in the required ring dimensions. Since the stated objective is a manufacturing route for internally cooled hollow shafts that can be adopted using existing workshop equipment, the use of commodity consumables is a deliberate design constraint rather than an oversight, and we have said so explicitly.

We have additionally added a description of the controlled surface texture (*R*_a_ 1.6 µm) specified for the ring mating faces, which was selected to make the interfacial contact condition reproducible across all ring pairs and all experimental runs.

### Comment 1.3 — Sample size of n = 5 and the reported 50 % improvement

**The manuscript reports a 50 % improvement in fatigue life, but the sample size (n = 5) is relatively small for reliable fatigue analysis. More replicates or statistical reliability analyses such as Weibull distribution or probabilistic fatigue assessment are recommended.**

A two-parameter Weibull analysis was in fact included in the original submission (Sections 2.6 and 3.4), but we recognise that its presentation gave it insufficient prominence: it appeared only as running text within the validation section, was absent from the Abstract, and was not reflected in any section heading or table. We are grateful to the Reviewer for drawing our attention to this, as the reliability analysis is central to the strength of our claim and should have been visible as such.

We have made four changes in response.

First, the analysis has been restructured into a dedicated **Section 3.5, “Reliability and Scatter Analysis”**, with a summary table (Table 8) giving shape and scale parameters, B10 and B50 characteristic lives, and coefficients of variation for both configurations. The key results are now also stated in the Abstract.

Second, we have added an explicit statement on the limitations of Weibull estimation at this sample size. Least-squares estimation of the shape parameter from five specimens is subject to substantial positive bias, and we now state that the absolute value of *β* = 26.69 should not be read as a reliable measure of scatter in its own right. The comparison between groups both estimated by an identical procedure from equal sample sizes remains informative, and it is corroborated by the directly computed coefficients of variation (3.7 % versus 8.3 %), which involve no distributional assumption.

Third, we have added a confidence interval for the difference in means, which we consider the most defensible summary of the effect. The difference of 163,629 cycles carries an approximate 95 % confidence interval of 129,800–197,400 cycles (Welch’s method, *df* = 7.92), corresponding to a ratio improvement spanning roughly 40 %–60 %. We now state that the magnitude of the improvement is established with reasonable confidence whereas its precise value is not.

Fourth, all instances of “50.0 %” have been replaced with “approximately 50 %” throughout, including the Abstract and Conclusions, and Conclusion 7 has been added to delimit the conditions under which the result was obtained.

Regarding the suggestion to increase the sample size: each validation specimen requires a full TIG pre-joining, WAAM deposition, double stress-relief annealing, and finish-machining cycle followed by a rotating bending test running to failure at 2900 rpm. We are not able to complete an expansion to *n* ≥ 10 per group within the revision period, and we have therefore stated this requirement explicitly as a recommendation for further work (Section 4.5) rather than presenting the current dataset as definitive.

### Comment 1.4 — Additional references

**Introduction and specific discussion can be enhanced by citing these references: (a) Effect of post weld thermal aging (PWTA) sensitization on micro-hardness and corrosion behavior of AISI 304 weld joints; (b) Sensitization studies on low cycle fatigue and cryogenic impact behavior of AISI 304 SS weldments; (c) Improvement in corrosion resistance of AISI 316L stainless steel weld cladding using GTA remelting technique; (d) A sensitization studies on the metallurgical and corrosion behavior of AISI 304 SS welds.**

We have reviewed all four suggested works. Three have been incorporated as references [49]–[51] and are cited in the Introduction at the point where the sensitivity of weld microstructure and properties to the imposed thermal cycle is established.

We found reference (c) particularly pertinent and have given it a correspondingly prominent role. That study applies a secondary GTA arc pass to an arc-deposited layer and reports a consequent modification of microstructure and an increase in microhardness a configuration closely analogous in principle to the TIG-plus-WAAM thermal sequence examined here, and one that supports our interpretation of the microhardness results now reported in Section 4.1.3. Reference (a), which likewise concerns microhardness of weld joints following thermal exposure, is a natural point of comparison for that section.

References (a) and (d) concern austenitic stainless steel weldments, where the controlling metallurgical mechanism is carbide precipitation and sensitisation rather than the ferrite–pearlite transformation relevant to our system. We have cited them as evidence for the general principle that a secondary thermal cycle applied to arc-deposited material can substantially alter its microstructure and mechanical performance, while noting explicitly in the text that the operative mechanism differs between the two alloy systems.

We were unable to locate verifiable bibliographic details journal, year, volume, or DOI for reference (b) despite extensive searching, and have therefore not cited it. We would be glad to include it if the Reviewer can supply the publication details.

### Comment 1.5 — Microstructural characterisation beyond OM and SEM

**The microstructural characterization is limited to OM and SEM observations. Additional characterization techniques such as EBSD, XRD, microhardness mapping, or TEM could provide deeper insight into phase evolution, grain orientation, and interfacial transformations.**

We accept this point and have implemented microhardness mapping, the technique among those suggested that we judged most informative for the specific question at issue. EBSD, XRD, and TEM are not available to us within the revision period, and we did not wish to claim capabilities we do not have.

A new **Section 2.8** describes the measurement protocol and a new **Section 4.1.3**, with **Fig 18** and **Table 9**, presents and interprets the results.

The measurement was designed as a controlled comparison rather than a survey. Two radial traverses were made on each specimen: traverse A1 positioned on a ring-to-ring interface and traverse A2 at the mid-ring position 1.8 mm away, on the same polished section, at the same radii, and prepared and measured under identical conditions. Any difference between them within a given specimen is therefore attributable to axial position relative to the interface and not to specimen preparation, instrument calibration, or bulk material variation.

The result forms a consistent four-way pattern within the substrate ring. At the mid-ring position the two configurations are statistically indistinguishable (176.4 versus 175.9 HV0.3, *p* = 0.85), and within the WAAM-only specimen there is no elevation at the interface relative to the mid-ring position (−1.9 HV0.3, *p* = 0.21). The TIG pre-joined specimen differs in both respects: its interface traverse is 8.2 HV0.3 harder than its own mid-ring traverse (*p* = 0.001) and 10.6 HV0.3 harder than the corresponding interface traverse in the WAAM-only specimen (*p* = 0.004). Two null comparisons and two positive comparisons together localise the effect: the TIG pass produces a measurable hardened zone confined to the interface region, and the substrate ring is otherwise unaffected by it.

A third traverse, run axially near the bore across two consecutive interfaces, shows the TIG pre-joined specimen to be uniformly 8.5 HV0.3 harder than the WAAM-only specimen (*p* < 0.001), consistent with the TIG pass having melted through the full ring wall the same conclusion reached independently from the metallographic sections.

The measurements also produced a result we did not anticipate and report in full. Within the WAAM deposit, where fatigue cracks initiated in every specimen, the two configurations are comparable (difference approximately 5 HV0.3, not consistently significant). Since Vickers hardness correlates principally with yield strength, this establishes that the two deposits are comparable in bulk mechanical properties at the crack initiation site, and therefore that the difference in fatigue behaviour cannot be attributed to one deposit simply being stronger than the other. We regard this as strengthening rather than weakening the argument, since it excludes an alternative explanation and localises the cause to the grain size and its distribution the microstructural feature that does differ, and the one that governs slip band formation.

We nevertheless agree that EBSD analysis of grain orientation and boundary character distribution across the interface, and XRD determination of phase fractions and residual stress, would deepen the mechanistic picture. Both are now named as specific priorities in Section 4.5.

### Comment 1.6 — Expansion of fracture analysis

**The fracture analysis should be expanded to include a detailed discussion of crack initiation sites, crack propagation regions, and final fracture zones.**

Section 4.2 has been restructured into three subsections following exactly this division: **4.2.1 Crack initiation sites**, **4.2.2 Crack propagation region**, and **4.2.3 Final fracture zone**. The figure annotations have been revised to use the same three terms.

The substantive content has also changed. Re-examination of the fracture surfaces established that cracks initiated at the outer surface in both configurations, and not at the interfacial features identified metallographically. Section 4.2.1 now presents a stress-based assessment supporting this: at the bore radius the bending stress amplitude is 116 MPa against 208.5 MPa at the outer fibre, and the stress intensity factor range associated with the unfused root is approximately 2–3 MPa·m^0.5^, well below the propagation threshold for ferritic–pearlitic steel. The unfused root therefore influences fatigue life through section loss and circumferential non-uniformity rather than by providing an initiation site.

Further additions include: quantification of the number and angular separation of initiation sites (one in the TIG-WAAM specimen; two, separated by approximately 90° of arc, in the WAAM-only specimen); an argument connecting multi-site initiation to the width of the grain size distribution rather than to interfacial defects; characterisation of the faceted propagation morphology in the WAAM-only specimen, whose facet dimensions correspond to the prior grain size and thereby link crack path directly to grain structure; description of the step generated where two independent crack fronts coalesced; and a distinction between interlayer lack of fusion within the deposit and incomplete root penetration at the ring interfaces, which are separate defect populations of different origin.

We also report a negative result. The boundary between the fatigue propagation region and the final overload region could not be delineated with sufficient confidence at the magnification employed for the relative extent of each region to be quantified, and we state this in the text rather than presenting areal estimates that the images do not support.

The section now closes with a synthesis describing the improvement as cumulative across all three stages rather than attributable to any single one.

### Comment 1.7 — Surface roughness and geometric irregularities

**The manuscript does not discuss the influence of surface roughness and the geometric irregularities inherent to WAAM processing, despite their known impact on fatigue performance.**

We agree that this is important, and on reflection we think the original manuscript was at fault for leaving the reader to infer our treatment of it rather than stating it. A dedicated passage has been added to Section 2.5.

The substance of our position is that surface roughness was a deliberately controlled variable rather than an omitted one. All specimens of both configurations were CNC turned and polished at the gauge section after deposition, removing the entire as-deposited layer together with its inter-bead waviness and stepped profile, and were verified by stylus profilometry to meet the surface finish requirement of ISO 1143. Holding surface roughness constant at a level where it is not the life-limiting feature is a necessary condition for attributing the observed life difference to the interfacial and microstructural effects of TIG pre-joining; had roughness been permitted to vary between the groups, the comparison would have been confounded. This is corroborated by the fractographic finding that initiation occurred at the machined surface in both configurations, at a location uncorrelated with the interfaces.

We have, however, taken the Reviewer’s point that this restricts the scope of our conclusions, and the revised text now states this explicitly: the reported lives characterise the machined condition, and for as-built WAAM shafts the absolute lives would be considerably lower while the relative benefit of TIG pre-joining could differ. Quantifying performance in the as-deposited condition, and determining the minimum machining allowance needed to recover machined-condition life, are now identified as directions for further work in Section 4.5.

### Comment 1.8 — Limitations and future work in the Conclusions

**The conclusions are generally supported by the presented results; however, they should include limitations of the current study and propose future research directions such as residual stress analysis, post-processing treatments, corrosion-fatigue evaluation, and multiaxial fatigue testing.**

Section 4.5 has been expanded to cover all four areas named, and a new Conclusion 7 has been added delimiting the conditions under which the reported improvement was established.

On residual stress: although both TIG pre-joining and WAAM deposition were followed by stress-relief annealing per ISO 17663, the residual stress state remaining at the interface was not measured. Since residual stress superimposes directly on the applied cyclic stress in rotating bending, its determination by X-ray diffraction or hole drilling would allow the mechanical and metallurgical contributions to the observed improvement to be separated a point we now regard as the single most significant open question arising from this work.

On post-processing: shot peening, deep rolling, and low-plasticity burnishing introduce compressive surface residual stress and could plausibly act in combination with, rather than in place of, the interfacial consolidation achieved by TIG pre-joining. This is of particular interest given the finding that crack initiation occurred at the outer surface in both configurations.

On corrosion-fatigue: hollow shafts carrying internal coolant are exposed to a corrosive medium at the bore surface, and the ring interfaces intersect that bore. The unfused root regions identified in the WAAM-only condition would present preferential paths for environmental attack, so evaluation in a representative coolant environment is necessary before service application.

On multiaxial fatigue: shafts in service experience combined bending and torsion, and testing under proportional and non-proportional loading would establish whether the benefit persists under a shear-dominated stress state, which is a more demanding condition for a transverse bonded plane.

We have additionally recorded the limitations of the new microhardness campaign (single traverses without replication; specimens representing group extremes rather than means; spatial autocorrelation along traverses affecting the interpretation of *p*-values), and one uninstrumented observation: a loosening of the clamping rod was noted on WAAM-only assemblies after deposition but not on TIG pre-joined assemblies. We offer this as an observation warranting dedicated investigation rather than as a supported finding.

### Comment 1.9 — References on the RSM method

**These references can be useful for RSM method used in the work: (a) Investigations on MWCNT embedded carbon/epoxy composite joints subjected to hygrothermal aging under bolt preloads; (b) Performance of Mechanical Joints Prepared from Carbon-Fiber-Reinforced Polymer Nanocomposites under Accelerated Environmental Aging.**

We thank the Reviewer for the suggestion. Having examined both works, we found them to address the performance of mechanical joints in composite material systems, where the process physics and the response variables differ substantially from arc-based metal deposition. Following the Academic Editor’s guidance that recommended works should be evaluated for relevance, we concluded that citing them in our treatment of RSM would not assist the reader.

We have, however, taken the underlying concern seriously that our treatment of RSM as a methodology was thinly supported and have strengthened the relevant passage of the Introduction with additional references on the application of RSM-CCD to arc welding and WAAM parameter optimisation specifically. We would of course be glad to include the suggested works if the Reviewer considers the connection closer than we have judged it to be.

### Comment 1.10 — Language

**Language: No (requires revision).**

The manuscript has undergone comprehensive language revision. Specific issues addressed: inconsistent British/American spelling, which appeared in both forms throughout the original; overlong sentences in the Discussion, several exceeding fifty words, which have been divided; inconsistent spacing around units and symbols; and several typographical errors, including a lowercase section heading, a malformed author affiliation marker, missing spaces after full stops, and an incomplete figure caption.

We have also corrected non-standard terminology in the figure annotations, replacing “instantaneous zone” with “final fracture zone” and adopting ISO 6520-1 terminology (“incomplete root penetration”) for the interfacial defect, distinguishing it from interlayer lack of fusion within the deposit.

## Response to Reviewer #2

We thank Reviewer #2 for a focused and practical set of comments, all of which have been implemented.

### Comment 2.1 — Title

**The title is confusing. Suggested: “Comparative Study of the Rotating Bending Fatigue Performance of Wire Arc Additively Manufactured Hollow Shafts”.**

We agree that the original title was awkwardly constructed and have adopted the Reviewer’s proposed formulation, with one addition:

Comparative Study of the Rotating Bending Fatigue Performance of Wire Arc Additively Manufactured Hollow Shafts **with and without TIG Pre-Joining**

Our reason for the addition is that the Reviewer’s title states that the study is comparative without indicating what is compared, and TIG pre-joining is the single independent variable distinguishing the two groups. We were also concerned that a title omitting it would reduce the discoverability of the paper. If the Reviewer prefers the shorter form as proposed, we will adopt it without reservation.

### Comment 2.2 — Abstract

**Delete the first two sentences of the Abstract.**

Both sentences have been removed. Because the first contained the only definition of the WAAM acronym, the opening has been reworded so that the expansion appears at first use in the new opening sentence. The Abstract has additionally been updated to include the Weibull results, the metallographic and microhardness findings, and the softened phrasing of the improvement figure discussed under Comment 1.3.

### Comment 2.3 — Introduction structure and citation density

**Avoid excessive paragraph fragmentation and avoid grouping many citations in a single sentence.**

The Introduction has been consolidated from seven paragraphs to five, organised as: technological context and application; the interfacial bonding challenge and the role of TIG pre-joining; methodological background; the study’s novelty relative to existing fatigue enhancement strategies; and the identified gap with the study objectives.

Grouped citations have been redistributed so that each reference supports a specific proposition. For instance, the passage previously reading “…are subjected to two successive thermal cycles one from the initial TIG pre-joining and the second from the multi-layer WAAM deposition [11–13]” has been expanded into separate sentences, each attributing a distinct claim to a single source. The same treatment has been applied to the other grouped citations, with a working limit of two references per sentence.

### Comment 2.4 — Equation numbering

**Equations must be numbered.**

All five equations are now numbered sequentially and right-aligned: Eq (1) heat input; Eq (2) section modulus; Eq (3) nominal bending stress amplitude; Eq (4) Benard’s median rank approximation, previously inline; and Eq (5) the fitted second-order response surface model, previously embedded in running text. All in-text references have been updated accordingly.

### Comment 2.5 — Scientific discussion in Section 3.1

**There is no strong scientific discussion of the fatigue life provided in section 3.1, which negatively impacts the study. It needs strong description under changing the process variable.**

We accept that the original Section 3.1 reported statistical outcomes without explaining them, and a substantial new subsection **3.1.1, “Physical interpretation of the parameter effects”**, has been added. Reviewer #1 raised a related concern regarding mechanistic depth, and we have written the new material to address both.

The subsection argues as follows. The statistical ranking of the four variables follows from their influence on heat input: deposition speed and current are the only variables that alter heat input directly, and speed does so over a wider relative range across the design space (1.5-fold versus 1.18-fold), which accounts for their respective *F*-values of 304.75 and 188.11 against an order of magnitude less for the geometric variables. The strongly negative quadratic coefficients for *v* and *I* reflect two competing damage mechanisms bracketing the design space: at low heat input the ring wall is incompletely melted, extending the unfused region at the bore and reducing the effective load-bearing section; at high heat input reduced cooling rates promote coarse columnar grains offering weaker barriers to early crack growth and lower resistance to initiation at the outer surface. Life is maximised where both are suppressed.

The significant *I* × *v* interaction follows directly, since current and speed occupy the numerator and denominator of the same heat input expression and their effects are therefore not additive, producing a ridge of constant heat input rather than an isolated peak. The absence of significant interactions involving *O* and *t* indicates that these act through mechanisms independent of the arc energy balance.

The treatment of ring thickness has been expanded considerably, as the original explanation in terms of thermal mass alone was incomplete. Because the stack length was held constant, the number of ring-to-ring interfaces is inversely proportional to *t*, falling five-fold across the design space; each interface carries its own heat-affected zone and also constitutes a thermal contact resistance impeding axial heat conduction. The positive linear coefficient for *t* therefore reflects three concurrent mechanisms increased thermal mass, reduced interface density, and reduced cumulative thermal contact resistance while the negative quadratic term reflects their saturation and reversal beyond *t* ≈ 3.6 mm.

## Corrections identified by the authors during revision

In the course of revision we verified every numerical value in the manuscript independently. We are grateful that this exercise identified several errors that had escaped our own checking, and we report them here in full.

1. **Contradictory statements regarding the 95 % prediction interval.** Section 3.4 stated that the mean validation result lay marginally below the lower bound of the prediction interval, while Conclusion 3 stated that it lay within the interval. The latter is correct: 490,973 cycles falls within 484,333–526,521 cycles. Section 3.4 has been corrected.
2. **Incorrect stress-to-yield-strength ratio.** Section 2.5 stated that the applied stress amplitude corresponded to approximately 30 %–50 % of the yield strength. For *σ*_a_ = 208.5 MPa and *σ*_y_ = 350–450 MPa the correct range is 46 %–60 %. Corrected, and the reference material for the quoted yield strength (the deposited ER70S-6, which forms the outer wall and governs behaviour at the outer fibre) is now stated explicitly.
3. **Mechanism count.** Section 4.3 announced five mechanisms and enumerated three. The section has been rewritten around three explicitly identified mechanisms.
4. **Inconsistent margin figure.** Section 4.4 referred to a 15 % margin between the mean validation life and the model prediction; the correct value is 2.9 %. Corrected.
5. **Inconsistent and incorrect grain size range.** The Abstract gave 55–90 µm for WAAM-only specimens against 50–90 µm elsewhere. Re-measurement established the correct range as 50–94 µm, which is now used consistently throughout.
6. **Incomplete data listing.** Section 3.4 listed three of the five individual TIG-WAAM validation results. All five are now given.
7. **Duplicate figure captions.** The captions for two SEM fractography figures were identical. Both have been rewritten to describe their distinct content.
8. **Table numbering.** The automatic numbering fields for all tables had failed in the submitted version, producing captions without numbers. All tables are now numbered explicitly.
9. **Figure assembly error.** In the longitudinal section figure, the two panels presented the WAAM-only specimen at two magnifications, whereas the caption described them as separate TIG-WAAM and WAAM-only specimens. The intended TIG-WAAM micrograph had been omitted during figure preparation. The figure has been reassembled with both configurations shown and the caption corrected.
10. **Transposed description of initiation site locations.** Section 4.2 described the initiation sites of the WAAM-only specimen using the location of those in the TIG-WAAM specimen. The description has been corrected and now specifies the angular separation between initiation sites rather than their absolute position, which carries no mechanistic information.

A number of minor typographical corrections have also been made.

We hope that the revisions address the reviewers’ concerns satisfactorily and that the manuscript is now suitable for publication in PLOS ONE. We would be glad to provide any further clarification or to undertake additional revision as required.

Yours sincerely,

**Tran Minh The Uyen** Corresponding author Ho Chi Minh City University of Technology and Engineering

E-mai: uyentmt@hcmute.edu.vn

on behalf of Van-Minh Nguyen, Pham Son Minh, and Hai Hung Pham
