# Supplementary material for: Comparative study of the rotating bending fatigue performance of wire arc additively manufactured hollow shafts with and without TIG pre-joining
Source: PLoS One. 2026 Sep 18;21(9):e0357916. doi: 10.1371/journal.pone.0357916 (PMC13588359; doi:10.1371/journal.pone.0357916)
Supplement: Peer review history

# Peer review: Decision Letter 0

**DOI:** 10.1371/journal.pone.0357916.r001

**Authors:** Gaurav Arora

4 Jun 2026

Dear Dr. The Uyen,

Thank you for submitting your manuscript to PLOS ONE. After careful consideration, we feel that it has merit but does not fully meet PLOS ONE’s publication criteria as it currently stands. Therefore, we invite you to submit a revised version of the manuscript that addresses the points raised during the review process.

==============================

**ACADEMIC EDITOR:**

Dear Authors,

The manuscript has been accessed, and major revision has been suggested by the respected reviewers.

Kindly address the comments and submit the revision timely.

==============================

Please submit your revised manuscript by Aug 03 2026 11:59PM. If you will need more time than this to complete your revisions, please reply to this message or contact the journal office at plosone@plos.org. When you're ready to submit your revision, log on to https://www.editorialmanager.com/pone/ and select the 'Submissions Needing Revision' folder to locate your manuscript file.

- A letter that responds to each point raised by the academic editor and reviewer(s). You should upload this letter as a separate file labeled 'Response to Reviewers'.
- A marked-up copy of your manuscript that highlights changes made to the original version. You should upload this as a separate file labeled 'Revised Manuscript with Track Changes'.
- An unmarked version of your revised paper without tracked changes. You should upload this as a separate file labeled 'Manuscript'.

If you would like to make changes to your financial disclosure, please include your updated statement in your cover letter. Guidelines for resubmitting your figure files are available below the reviewer comments at the end of this letter.

If applicable, we recommend that you deposit your laboratory protocols in protocols.io to enhance the reproducibility of your results. Protocols.io assigns your protocol its own identifier (DOI) so that it can be cited independently in the future. For instructions see: https://journals.plos.org/plosone/s/submission-guidelines#loc-laboratory-protocols. Additionally, PLOS ONE offers an option for publishing peer-reviewed Lab Protocol articles, which describe protocols hosted on protocols.io. Read more information on sharing protocols at https://plos.org/protocols?utm_medium=editorial-email&utm_source=authorletters&utm_campaign=protocols.

As the corresponding author, your ORCID iD is verified in the submission system and will appear in the published article. PLOS supports the use of ORCID, and we encourage all coauthors to register for an ORCID iD and use it as well. Please encourage your coauthors to verify their ORCID iD within the submission system before final acceptance, as unverified ORCID iDs will not appear in the published article. *Only*  the individual author can complete the verification step; PLOS staff *cannot*  verify ORCID iDs on behalf of authors.

We look forward to receiving your revised manuscript.

Kind regards,

Gaurav Arora, Ph.D

Academic Editor

PLOS One

Journal Requirements:

When submitting your revision, we need you to address these additional requirements.

1. Please ensure that your manuscript meets PLOS ONE's style requirements, including those for file naming. The PLOS ONE style templates can be found at

https://journals.plos.org/plosone/s/file?id=wjVg/PLOSOne_formatting_sample_main_body.pdf and

https://journals.plos.org/plosone/s/file?id=ba62/PLOSOne_formatting_sample_title_authors_affiliations.pdf

2. We note that the grant information you provided in the ‘Funding Information’ and ‘Financial Disclosure’ sections do not match.

When you resubmit, please ensure that you provide the correct grant numbers for the awards you received for your study in the ‘Funding Information’ section.

3. Thank you for stating the following financial disclosure:

“The author(s) received no specific funding for this work.”

At this time, please address the following queries:

a)        Please clarify the sources of funding (financial or material support) for your study. List the grants or organizations that supported your study, including funding received from your institution.

b)        State what role the funders took in the study. If the funders had no role in your study, please state: “The funders had no role in study design, data collection and analysis, decision to publish, or preparation of the manuscript.”

c)        If any authors received a salary from any of your funders, please state which authors and which funders.

d)        If you did not receive any funding for this study, please state: “The authors received no specific funding for this work.”

Please include your amended statements within your cover letter; we will change the online submission form on your behalf.

4. If the reviewer comments include a recommendation to cite specific previously published works, please review and evaluate these publications to determine whether they are relevant and should be cited. There is no requirement to cite these works unless the editor has indicated otherwise.

Additional Editor Comments :

Dear Authors,

The manuscript has been accessed, and major revision has been suggested by the respected reviewers.

Kindly address the comments and submit the revision timely.

[Note: HTML markup is below. Please do not edit.]

Reviewers' comments:

Reviewer's Responses to Questions

**Comments to the Author**

1. Is the manuscript technically sound, and do the data support the conclusions?

Reviewer #1: Partly

Reviewer #2: Yes

**********

2. Has the statistical analysis been performed appropriately and rigorously?

Reviewer #1: N/A

Reviewer #2: Yes

**********

3. Have the authors made all data underlying the findings in their manuscript fully available?

The PLOS Data policy

Reviewer #1: Yes

Reviewer #2: Yes

**********

4. Is the manuscript presented in an intelligible fashion and written in standard English?

Reviewer #1: No

Reviewer #2: Yes

**********

Reviewer #1: Reviewer Comments

- The novelty of incorporating TIG pre-joining prior to WAAM deposition should be more clearly established against existing studies on WAAM fatigue enhancement techniques.

- The manuscript lacks sufficient justification for selecting ER70S-6 wire and CCT34 low-carbon steel substrate rings.

- The manuscript reports a 50% improvement in fatigue life, but the sample size (n = 5) is relatively small for reliable fatigue analysis. More replicates or statistical reliability analyses such as Weibull distribution or probabilistic fatigue assessment are recommended.

- Introduction and specific discussion can be enhanced by citing these references:

a. Effect of post weld thermal aging (PWTA) sensitization on micro-hardness and corrosion behavior of AISI 304 weld joints.

b. Sensitization studies on low cycle fatigue and cryogenic impact behavior of AISI 304 SS weldments.

c. Improvement in corrosion resistance of AISI 316L stainless steel weld cladding using GTA remelting technique.

d. A sensitization studies on the metallurgical and corrosion behavior of AISI 304 SS welds.

- The microstructural characterization is limited to OM and SEM observations. Additional characterization techniques such as EBSD, XRD, microhardness mapping, or TEM could provide deeper insight into phase evolution, grain orientation, and interfacial transformations.

- The fracture analysis should be expanded to include a detailed discussion of crack initiation sites, crack propagation regions, and final fracture zones.

- These references can be useful for RSM method used in the work.

a. Investigations on MWCNT embedded carbon/epoxy composite joints subjected to hygrothermal aging under bolt preloads

b. Performance of Mechanical Joints Prepared from Carbon-Fiber-Reinforced Polymer Nanocomposites under Accelerated Environmental Aging

- The manuscript does not discuss the influence of surface roughness and geometric irregularities inherent to WAAM processing, despite their known impact on fatigue performance.

- The conclusions are generally supported by the presented results; however, they should include limitations of the current study and propose future research directions such as residual stress analysis, post-processing treatments, corrosion-fatigue evaluation, and multiaxial fatigue testing.

Reviewer #2: This study describes the development and investigations of rotating bending fatigue performance, manufactured by wire arc additive manufacturing. Following minor modifications are required.

1. Title creates confusion. In my opinion the title should be modified to “Comparative Study of the Rotating Bending Fatigue Performance of Wire Arc Additively Manufactured Hollow Shafts”.

2. First two sentences in the abstract should be removed.

3. Avoid many paragraphs breakage and cumulative citation for single sentence in introduction section.

4. Equations should be numbered.

5. There is no strong scientific discussion of the Fatigue life was provided in section 3.1 which negatively impact the study. It need string description under changing the process variable.

**********

what does this mean?). If published, this will include your full peer review and any attached files.

If you choose “no”, your identity will remain anonymous but your review may still be made public.

**Do you want your identity to be public for this peer review?**  For information about this choice, including consent withdrawal, please see our Privacy Policy

Reviewer #1: No

Reviewer #2: No

**********

[NOTE: If reviewer comments were submitted as an attachment file, they will be attached to this email and accessible via the submission site. Please log into your account, locate the manuscript record, and check for the action link "View Attachments". If this link does not appear, there are no attachment files.]

To ensure your figures meet our technical requirements, please review our figure guidelines: https://journals.plos.org/plosone/s/figures

You may also use PLOS’s free figure tool, NAAS, to help you prepare publication quality figures: https://journals.plos.org/plosone/s/figures#loc-tools-for-figure-preparation.

NAAS will assess whether your figures meet our technical requirements by comparing each figure against our figure specifications.

# Author comment: Author response to Decision Letter 1

**DOI:** 10.1371/journal.pone.0357916.r002

3 Aug 2026

Response to Reviewer #1

We are grateful to Reviewer #1 for a demanding and detailed assessment. The points raised regarding novelty, material justification, statistical inference, microstructural characterisation, and fractographic depth have materially strengthened the manuscript.

Comment 1.1 — Novelty relative to existing work on WAAM fatigue improvement

The novelty of incorporating TIG pre-joining prior to WAAM deposition should be more clearly established against existing studies on WAAM fatigue enhancement techniques.

We accept that the original framing did not make the distinction clear, and we have rewritten the closing portion of the Introduction accordingly.

The revised text makes the following argument. Existing strategies for improving WAAM fatigue performance post-deposition heat treatment, mechanical surface treatments such as shot peening or machining, and in-process interventions such as interpass rolling all operate on a monolithic deposit built on a simple flat or bar substrate. The fatigue-critical features they address are consequently internal to the deposit: porosity, columnar grain morphology, or as-built surface waviness.

The configuration studied here is different in kind. Building a hollow shaft on a stack of discrete substrate rings introduces a fatigue-critical feature absent from monolithic WAAM entirely: a series of transverse ring-to-ring interfaces distributed along the component axis. Because deposition proceeds from the outer surface inward, the root of each interface lies at the bore and is the last region reached by the molten pool, so that any incompleteness in fusion is concentrated precisely where the process has least control. Neither post-deposition heat treatment nor surface treatment can remedy such a discontinuity, because it lies beneath the deposit and is created before deposition begins. It can only be eliminated at the assembly stage, which is the specific function of the TIG pre-joining step.

We have also strengthened the survey of prior work to demonstrate that this configuration is not covered by existing literature. Ermakova et al. [28] characterised uniaxial and multiaxial fatigue of WAAM ER70S-6 but tested solid bar specimens from a monolithic wall; Fang et al. [26] examined high-cycle fatigue of WAAM carbon steel plates. In neither case did the specimen contain a substrate interface. Work on hollow and tubular WAAM geometries has concentrated on deposition strategy and dimensional accuracy rather than cyclic performance, and studies of WAAM on structured substrates have reported static properties without extending to fatigue [29,30].

We would add that framing the novelty in terms of the geometry and substrate architecture, rather than in terms of TIG welding as such, is in our view the accurate description of the contribution, and we thank the Reviewer for prompting us to state it precisely.

Comment 1.2 — Justification for the choice of ER70S-6 wire and CCT34 substrate rings

The manuscript lacks sufficient justification for selecting ER70S-6 wire and CCT34 low-carbon steel substrate rings.

A dedicated justification has been added to Section 2.1, together with a new table of nominal chemical compositions (Table 1).

The rationale rests on three grounds. Metallurgically, the bond between the deposited bead and the substrate ring is formed by arc melting and solidification and is therefore, in essence, a fusion weld; its integrity is governed by the same factors that control weldability in conventional arc welding of low-carbon steels, namely compatibility of composition, carbon equivalent, and solidification range. ER70S-6 and CCT34 are both low-carbon, low-alloy steels with closely comparable carbon and manganese contents, so dilution at the interface produces a fusion zone spanning two similar parent compositions rather than a dissimilar-metal gap. This avoids the hard martensitic transition layers, sharp hardness discontinuities, and differential thermal contraction associated with dissimilar-alloy combinations, all of which are known promoters of interfacial crack initiation under cyclic loading. The microhardness profiles now reported in Section 4.1.3 provide direct experimental confirmation: no sharp hardness discontinuity was observed at the substrate–deposit boundary in either configuration.

Functionally, the elevated silicon and manganese content of ER70S-6 provides strong deoxidation, which matters when depositing onto a ground ring surface that may retain a thin oxide film, and the wire exhibits stable bead formation across a wide current range a prerequisite for the broad parameter window explored here.

Practically, ER70S-6 in 1.2 mm diameter is the most widely available and lowest-cost solid welding wire in the Vietnamese market and is compatible with standard MIG power sources, while CCT34 seamless tube is a commodity product available in the required ring dimensions. Since the stated objective is a manufacturing route for internally cooled hollow shafts that can be adopted using existing workshop equipment, the use of commodity consumables is a deliberate design constraint rather than an oversight, and we have said so explicitly.

We have additionally added a description of the controlled surface texture (Ra 1.6 µm) specified for the ring mating faces, which was selected to make the interfacial contact condition reproducible across all ring pairs and all experimental runs.

Comment 1.3 — Sample size of n = 5 and the reported 50 % improvement

The manuscript reports a 50 % improvement in fatigue life, but the sample size (n = 5) is relatively small for reliable fatigue analysis. More replicates or statistical reliability analyses such as Weibull distribution or probabilistic fatigue assessment are recommended.

A two-parameter Weibull analysis was in fact included in the original submission (Sections 2.6 and 3.4), but we recognise that its presentation gave it insufficient prominence: it appeared only as running text within the validation section, was absent from the Abstract, and was not reflected in any section heading or table. We are grateful to the Reviewer for drawing our attention to this, as the reliability analysis is central to the strength of our claim and should have been visible as such.

We have made four changes in response.

First, the analysis has been restructured into a dedicated Section 3.5, “Reliability and Scatter Analysis”, with a summary table (Table 8) giving shape and scale parameters, B10 and B50 characteristic lives, and coefficients of variation for both configurations. The key results are now also stated in the Abstract.

Second, we have added an explicit statement on the limitations of Weibull estimation at this sample size. Least-squares estimation of the shape parameter from five specimens is subject to substantial positive bias, and we now state that the absolute value of β = 26.69 should not be read as a reliable measure of scatter in its own right. The comparison between groups both estimated by an identical procedure from equal sample sizes remains informative, and it is corroborated by the directly computed coefficients of variation (3.7 % versus 8.3 %), which involve no distributional assumption.

Third, we have added a confidence interval for the difference in means, which we consider the most defensible summary of the effect. The difference of 163,629 cycles carries an approximate 95 % confidence interval of 129,800–197,400 cycles (Welch’s method, df = 7.92), corresponding to a ratio improvement spanning roughly 40 %–60 %. We now state that the magnitude of the improvement is established with reasonable confidence whereas its precise value is not.

Fourth, all instances of “50.0 %” have been replaced with “approximately 50 %” throughout, including the Abstract and Conclusions, and Conclusion 7 has been added to delimit the conditions under which the result was obtained.

Regarding the suggestion to increase the sample size: each validation specimen requires a full TIG pre-joining, WAAM deposition, double stress-relief annealing, and finish-machining cycle followed by a rotating bending test running to failure at 2900 rpm. We are not able to complete an expansion to n ≥ 10 per group within the revision period, and we have therefore stated this requirement explicitly as a recommendation for further work (Section 4.5) rather than presenting the current dataset as definitive.

Comment 1.4 — Additional references

Introduction and specific discussion can be enhanced by citing these references: (a) Effect of post weld thermal aging (PWTA) sensitization on micro-hardness and corrosion behavior of AISI 304 weld joints; (b) Sensitization studies on low cycle fatigue and cryogenic impact behavior of AISI 304 SS weldments; (c) Improvement in corrosion resistance of AISI 316L stainless steel weld cladding using GTA remelting technique; (d) A sensitization studies on the metallurgical and corrosion behavior of AISI 304 SS welds.

We have reviewed all four suggested works. Three have been incorporated as references [49]–[51] and are cited in the Introduction at the point where the sensitivity of weld microstructure and properties to the imposed thermal cycle is established.

We found reference (c) particularly pertinent and have given it a correspondingly prominent role. That study applies a secondary GTA arc pass to an arc-deposited layer and reports a consequent modification of microstructure and an increase in microhardness a configuration closely analogous in principle to the TIG-plus-WAAM thermal sequence examined here, and one that supports our interpretation of the microhardness results now reported in Section 4.1.3. Reference (a), which likewise concerns microhardness of weld joints following thermal exposure, is a natural point of comparison for that section.

References (a) and (d) concern austenitic stainless steel weldments, where the controlling metallurgical mechanism is carbide precipitation and sensitisation rather than the ferrite–pearlite transformation relevant to our system. We have cited them as evidence for the general principle that a secondary thermal cycle applied to arc-deposited material can substantially alter its microstructure and mechanical performance, while noting explicitly in the text that the operative mechanism differs between the two alloy systems.

We were unable to locate verifiable bibliographic details journal, year, volume, or DOI for reference (b) despite extensive searching, and have therefore not cited it. We would be glad to include it if the Reviewer can supply the publication details.

Comment 1.5 — Microstructural characterisation beyond OM and SEM

The microstructural characterization is limited to OM and SEM observations. Additional characterization techniques such as EBSD, XRD, microhardness mapping, or TEM could provide deeper insight into phase evolution, grain orientation, and interfacial transformations.

We accept this point and have implemented microhardness mapping, the technique among those suggested that we judged most informative for the specific question at issue. EBSD, XRD, and TEM are not available to us within the revision period, and we did not wish to claim capabilities we do not have.

A new Section 2.8 describes the measurement protocol and a new Section 4.1.3, with Fig 18 and Table 9, presents and interprets the results.

The measurement was designed as a controlled comparison rather than a survey. Two radial traverses were made on each specimen: traverse A1 positioned on a ring-to-ring interface and traverse A2 at the mid-ring position 1.8 mm away, on the same polished section, at the same radii, and prepared and measured under identical conditions. Any difference between them within a given specimen is therefore attributable to axial position relative to the interface and not to specimen preparation, instrument calibration, or bulk material variation.

The result forms a consistent four-way pattern within the substrate ring. At the mid-ring position the two configurations are statistically indistinguishable (176.4 versus 175.9 HV0.3, p = 0.85), and within the WAAM-only specimen there is no elevation at the interface relative to the mid-ring position (−1.9 HV0.3, p = 0.21). The TIG pre-joined specimen differs in both respects: its interface traverse is 8.2 HV0.3 harder than its own mid-ring traverse (p = 0.001) and 10.6 HV0.3 harder than the corresponding interface traverse in the WAAM-only specimen (p = 0.004). Two null comparisons and two positive comparisons together localise the effect: the TIG pass produces a measurable hardened zone confined to the interface region, and the substrate ring is otherwise unaffected by it.

A third traverse, run axially near the bore across two consecutive interfaces, shows the TIG pre-joined specimen to be uniformly 8.5 HV0.3 harder than the WAAM-only specimen (p < 0.001), consistent with the TIG pass having melted through the full ring wall the same conclusion reached independently from the metallographic sections.

The measurements also produced a result we did not anticipate and report in full. Within the WAAM deposit, where fatigue cracks initiated in every specimen, the two configurations are comparable (difference approximately 5 HV0.3, not consistently significant). Since Vickers hardness correlates principally with yield strength, this establishes that the two deposits are comparable in bulk mechanical properties at the crack initiation site, and therefore that the difference in fatigue behaviour cannot be attributed to one deposit simply being stronger than the other. We regard this as strengthening rather than weakening the argument, since it excludes an alternative explanation and localises the cause to the grain size and its distribution the microstructural feature that does differ, and the one that governs slip band formation.

We nevertheless agree that EBSD analysis of grain orientation and boundary character distribution across the interface, and XRD determination of phase fractions and residual stress, would deepen the mechanistic picture. Both are now named as specific priorities in Section 4.5.

Comment 1.6 — Expansion of fracture analysis

The fracture analysis should be expanded to include a detailed discussion of crack initiation sites, crack propagation regions, and final fracture zones.

Section 4.2 has been restructured into three subsections following exactly this division: 4.2.1 Crack initiation sites, 4.2.2 Crack propagation region, and 4.2.3 Final fracture zone. The figure annotations have been revised to use the same three terms.

The substantive content has also changed. Re-examination of the fracture surfaces established that cracks initiated at the outer surface in both configurations, and not at the interfacial features identified metallographically. Section 4.2.1 now presents a stress-based assessment supporting this: at the bore radius the bending stress amplitude is 116 MPa against 208.5 MPa at the outer fibre, and the stress intensity factor range associated with the unfused root is approximately 2–3 MPa·m0.5, well below the propagation threshold for ferritic–pearlitic steel. The unfused root therefore influences fatigue life through section loss and circumferential non-uniformity rather than by providing an initiation site.

Further additions include: quantification of the number and angular separation of initiation sites (one in the TIG-WAAM specimen; two, separated by approximately 90° of arc, in the WAAM-only specimen); an argument connecting multi-site initiation to the width of the grain size distribution rather than to interfacial defects; characterisation of the faceted propagation morphology in the WAAM-only specimen, whose facet dimensions correspond to the prior grain size and thereby link crack path directly to grain structure; description of the step generated where two independent crack fronts coalesced; and a distinction between interlayer lack of fusion within the deposit and incomplete root penetration at the ring interfaces, which are separate defect populations of different origin.

We also report a negative result. The boundary between the fatigue propagation region and the final overload region could not be delineated with sufficient confidence at the magnification employed for the relative extent

Attachment Submitted filename: Response_to_Editor.docx

# Peer review: Decision Letter 1

**DOI:** 10.1371/journal.pone.0357916.r003

**Authors:** Gaurav Arora

24 Aug 2026

<p>Comparative Study of the Rotating Bending Fatigue Performance of Wire Arc Additively Manufactured Hollow Shafts with and without TIG Pre-Joining

PONE-D-26-20584R1

Dear Dr. The Uyen,

We’re pleased to inform you that your manuscript has been judged scientifically suitable for publication and will be formally accepted for publication once it meets all outstanding technical requirements.

Within one week, you’ll receive an e-mail detailing the required amendments. When these have been addressed, you’ll receive a formal acceptance letter and your manuscript will be scheduled for publication.

An invoice will be generated when your article is formally accepted. Please note, if your institution has a publishing partnership with PLOS and your article meets the relevant criteria, all or part of your publication costs will be covered. Please make sure your user information is up-to-date by logging into Editorial Manager at Editorial Manager® and clicking the ‘Update My Information' link at the top of the page. For questions related to billing, please contact billing support.

If your institution or institutions have a press office, please notify them about your upcoming paper to help maximize its impact. If they’ll be preparing press materials, please inform our press team as soon as possible -- no later than 48 hours after receiving the formal acceptance. Your manuscript will remain under strict press embargo until 2 pm Eastern Time on the date of publication. For more information, please contact onepress@plos.org.

Kind regards,

Academic Editor

PLOS One

Additional Editor Comments (optional):

The acceptance has been given by the respected reviewers.

Reviewers' comments:

Reviewer's Responses to Questions

**Comments to the Author**

Reviewer #1: All comments have been addressed

Reviewer #2: All comments have been addressed

**********

2. Is the manuscript technically sound, and do the data support the conclusions?

Reviewer #1: Yes

Reviewer #2: Yes

**********

3. Has the statistical analysis been performed appropriately and rigorously?

Reviewer #1: N/A

Reviewer #2: Yes

**********

4. Have the authors made all data underlying the findings in their manuscript fully available?

The PLOS Data policy

Reviewer #1: Yes

Reviewer #2: Yes

**********

5. Is the manuscript presented in an intelligible fashion and written in standard English?

Reviewer #1: Yes

Reviewer #2: Yes

**********

Reviewer #1: The manuscript has been carefully revised and is suitable for acceptance. The authors have addressed all major concerns satisfactorily.

Reviewer #2: The revised version is acceptable. The authors have successfully addressed the response to the comments from reviewer.

**********

what does this mean?). If published, this will include your full peer review and any attached files.

If you choose “no”, your identity will remain anonymous but your review may still be made public.

**Do you want your identity to be public for this peer review?**  For information about this choice, including consent withdrawal, please see our Privacy Policy

Reviewer #1: No

Reviewer #2: No

**********

# Editor report: Acceptance letter

**DOI:** 10.1371/journal.pone.0357916.r004

**Authors:** Gaurav Arora

PONE-D-26-20584R1

PLOS One

Dear Dr. The Uyen,

I'm pleased to inform you that your manuscript has been deemed suitable for publication in PLOS One. Congratulations! Your manuscript is now being handed over to our production team.

At this stage, our production department will prepare your paper for publication. This includes ensuring the following:

* All references, tables, and figures are properly cited

* All relevant supporting information is included in the manuscript submission,

* There are no issues that prevent the paper from being properly typeset

You will receive further instructions from the production team, including instructions on how to review your proof when it is ready. Please keep in mind that we are working through a large volume of accepted articles, so please give us a few days to review your paper and let you know the next and final steps.

Lastly, if your institution or institutions have a press office, please let them know about your upcoming paper now to help maximize its impact. If they'll be preparing press materials, please inform our press team within the next 48 hours. Your manuscript will remain under strict press embargo until 2 pm Eastern Time on the date of publication. For more information, please contact onepress@plos.org.

You will receive an invoice from PLOS for your publication fee after your manuscript has reached the completed accept phase. If you receive an email requesting payment before acceptance or for any other service, this may be a phishing scheme. Learn how to identify phishing emails and protect your accounts at https://explore.plos.org/phishing.

If we can help with anything else, please email us at customercare@plos.org.

Thank you for submitting your work to PLOS One and supporting open access.

Kind regards,

PLOS One Editorial Office Staff

on behalf of

Dr. Gaurav Arora

Academic Editor

PLOS One
